# Translocation of a Bak C-Terminus Mutant from Cytosol to Mitochondria to Mediate Cytochrome *c* Release: Implications for Bak and Bax Apoptotic Function

**DOI:** 10.1371/journal.pone.0031510

**Published:** 2012-03-19

**Authors:** Pedro Eitz Ferrer, Paul Frederick, Jacqueline M. Gulbis, Grant Dewson, Ruth M. Kluck

**Affiliations:** 1 The Walter and Eliza Hall Institute of Medical Research, Parkville, Victoria, Australia; 2 Department of Medical Biology, The University of Melbourne, Parkville, Victoria, Australia; University of Illinois at Chicago, United States of America

## Abstract

**Background:**

One of two proapoptotic Bcl-2 proteins, Bak or Bax, is required to permeabilize the mitochondrial outer membrane during apoptosis. While Bax is mostly cytosolic and translocates to mitochondria following an apoptotic stimulus, Bak is constitutively integrated within the outer membrane. Membrane anchorage occurs via a C-terminal transmembrane domain that has been studied in Bax but not in Bak, therefore what governs their distinct subcellular distribution is uncertain. In addition, whether the distinct subcellular distributions of Bak and Bax contributes to their differential regulation during apoptosis remains unclear.

**Methodology/Principal Findings:**

To gain insight into Bak and Bax targeting to mitochondria, elements of the Bak C-terminus were mutated, or swapped with those of Bax. Truncation of the C-terminal six residues (C-segment) or substitution of three basic residues within the C-segment destabilized Bak. Replacing the Bak C-segment with that from Bax rescued stability and function, but unexpectedly resulted in a semi-cytosolic protein, termed Bak/BaxCS. When in the cytosol, both Bax and Bak/BaxCS sequestered their hydrophobic transmembrane domains in their hydrophobic surface groove. Upon apoptotic signalling, Bak/BaxCS translocated to the mitochondrial outer membrane, inserted its transmembrane domain, oligomerized, and released cytochrome *c*. Despite this Bax-like subcellular distribution, Bak/BaxCS retained Bak-like regulation following targeting of Mcl-1.

**Conclusions/Significance:**

Residues in the C-segment of Bak and of Bax contribute to their distinct subcellular localizations. That a semi-cytosolic form of Bak, Bak/BaxCS, could translocate to mitochondria and release cytochrome *c* indicates that Bak and Bax share a conserved mode of activation. In addition, the differential regulation of Bak and Bax by Mcl-1 is predominantly independent of the initial subcellular localizations of Bak and Bax.

## Introduction

The intrinsic or mitochondrial pathway of apoptosis is regulated by the Bcl-2 protein family, with two members, Bak and Bax, required to permeabilize the mitochondrial outer membrane (OM) [1], [2]. During OM permeabilization, Bak and Bax undergo significant conformation change involving exposure of N-terminal epitopes and homo-oligomerization [3], [4], [5], [6], [7] to form an as yet undefined pore. Bak conformation change also involves transient exposure of the BH3 domain that then binds to the hydrophobic groove of another activated Bak molecule to form symmetric dimers [8], [9], [10], with the same process also evident for Bax [11], [12], [13], [14]. Symmetric dimers of Bak and of Bax can then be linked by an α6∶α6 interface into higher order oligomers that likely constitute the apoptotic pore [8], [12].

Bak and Bax are regulated by other Bcl-2 family members. They are activated by direct binding of BH3-only proteins (e.g. Bim and tBid), and sequestered by binding to prosurvival proteins (e.g. Mcl-1 and Bcl-2) [15], [16], [17], [18], [19], [20]. Specific binding results in Bak being guarded mainly by Bcl-x_L_, Mcl-1 and A1, while Bax is countered mainly by Bcl-2, Bcl-x_L_, Bcl-w and A1 [20], [21], [22], [23]. This specific binding can result in either Bak or Bax preferentially driving apoptosis [24], [25]. For example, Bak-driven apoptosis can be initiated by loss of the relatively labile Mcl-1 and Bcl-x_L_ following UV, actinomycin D or cycloheximide [20],[26].

Bak and Bax contain a C-terminal hydrophobic region that inserts as a transmembrane (TM) domain into the mitochondrial OM. The C-termini of Bak and Bax can target GFP to mitochondria [27], [28], and their truncation in the native proteins can block membrane insertion and proapoptotic function [27], [28], [29], [30], [31]. Two or more basic residues in the extreme C-terminus (i.e. within the C-segment) may assist insertion of the TM domain across the OM, as observed for other mitochondrial tail-anchored proteins [30], [32], [33]. Whether the TM domain inserts spontaneously across the mitochondrial membrane remains controversial [27], [28], however peptides equivalent to the C-termini of Bak (24 residues) and of Bax (24 residues) can integrate into model membranes in the absence of chaperones or receptors [34], [35].

Bak is integrated in the OM in healthy cells, whereas Bax is largely cytosolic until its translocation to mitochondria after apoptotic signalling [36]. A portion of Bax that is peripherally attached to mitochondria in healthy cells can retrotranslocate upon binding Bcl-x_L_ [37]. Cytosolic Bax is proposed to sequester its TM domain in a hydrophobic surface groove through an interaction involving hydrogen bonding between S184 in the TM domain and D98 in the groove [30], [38]. While others have examined how the C-termini of Bax, Bcl-x_L_, and Bcl-2 control mitochondrial targeting [30], [31], [39], [40], this has not been examined for Bak.

To understand how Bak is targeted to mitochondria, and to address whether differences in Bak and Bax localization might contribute to their differential regulation, we mutated the C-terminus of Bak. Removing the C-segment (C-terminal six residues), or the basic residues within, decreased mitochondrial targeting and protein stability, thereby decreasing proapoptotic function. Notably, replacing the C-segment of Bak with that from Bax converted Bak to a relatively stable, semi-cytosolic protein (named Bak/BaxCS) that could translocate to mitochondria. Translocation of both Bak/BaxCS and Bax following apoptotic signalling suggests similar activation mechanisms for Bak and Bax. Furthermore, the semi-cytosolic localization of Bak/BaxCS did not alter its regulation by Mcl-1.

## Results

### The Bak C-segment is required for stability, mitochondrial targeting and proapoptotic function

**Figure 1:**
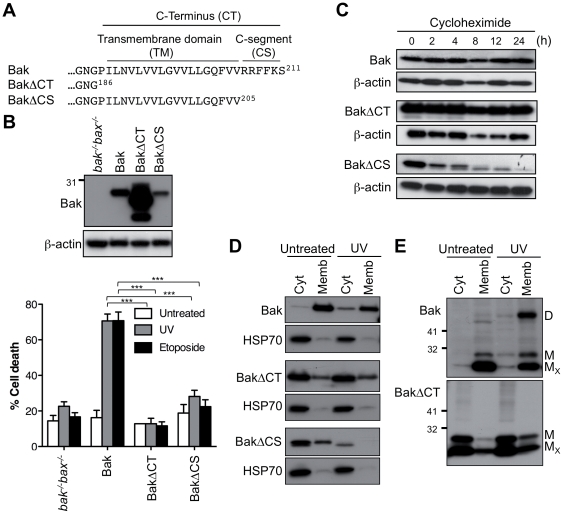
The Bak C-segment is essential for stability, membrane targeting and proapoptotic function. (**A**) C-terminal sequence of Bak variants truncated at the C-terminus. The C-terminus (CT) contains a hydrophobic transmembrane (TM) domain and a hydrophilic C-segment (CS). (**B**) Truncation of the Bak C-terminus or C-segment blocks proapoptotic function. *bak^−/−^bax^−/−^*MEFs expressing Bak, BakΔCT and BakΔCS were left untreated, or treated with UV or etoposide for 24 h. Percentage cell death is expressed as the mean ± SEM from three independent experiments. Statistical significance for treatment when compared to Bak; ***p<0.001. Upper panel is a western blot of cell lysates immunoblotted for Bak, and for β-actin as a loading control. (**C**) Truncation of the C-segment reduces half-life. Cells from (B) were incubated with cycloheximide for up to 24 h and cell lysates immunoblotted for Bak, and for β-actin as a loading control. Note that due to low expression of BakΔCS, 4-fold total protein was loaded onto the gels. (**D**) Truncation of the Bak C-terminus prevents membrane targeting. Cells were left untreated or treated with UV, separated into cytosolic and membrane fractions, and immunoblotted for Bak and the cytosolic marker HSP70. (**E**) Bak lacking the C-terminus fails to undergo conformation change and oligomerization in response to UV. Cells treated as in (D) were exposed to oxidant (CuPhe), separated into cytosolic and membrane fractions, run on non-reducing SDS-PAGE and immunoblotted for Bak. M_X_, non-activated intramolecular cross-linked monomer; M, non-crosslinked monomer; D, intermolecular crosslinked dimers. Results are representative of two or more independent experiments.

To identify elements in the Bak C-terminus that are required for OM targeting and integration, the whole C-terminus or just the C-segment was truncated (BakΔCT, BakΔCS; Figure 1A). Following stable expression in *bak^−/−^bax^−/−^* MEFs, both truncated proteins failed to mediate cytochrome *c* release and apoptosis after exposure to UV or etoposide (Figure 1B and Figure S1). BakΔCT was relatively stable, as protein expression levels were very high, and half-life as assessed by incubation in cycloheximide was similar to that of Bak (Figures 1B and C). In contrast, BakΔCS was unstable as indicated by low expression level, low half-life, and degradation following UV treatment (Figures 1B, C, D), perhaps due to an exposed TM domain targeting the protein for degradation. The truncated proteins were inefficient at targeting membranes both before and after apoptotic signalling (Figures 1D and E). In Bax, truncation of the C-terminus (23 residues) or C-segment (^188^WKKMG^192^) also blocked mitochondrial translocation and function (data not shown), as reported previously [27], [30], [31]. Thus, the C-termini, including the C-segments, are required for targeting stably expressed Bak and Bax to mitochondria.

Notably, the Bak C-terminus was also necessary for apoptotic conformation change and oligomerization, as BakΔCT essentially remained in the non-activated conformation, and failed to dimerize following UV treatment (Figure 1E). Moreover, BakΔCT failed to change conformation following addition of the proapoptotic BH3-only protein tBid to cell extracts [8].

### Basic residues in the Bak C-segment govern stability and function

**Figure 2:**
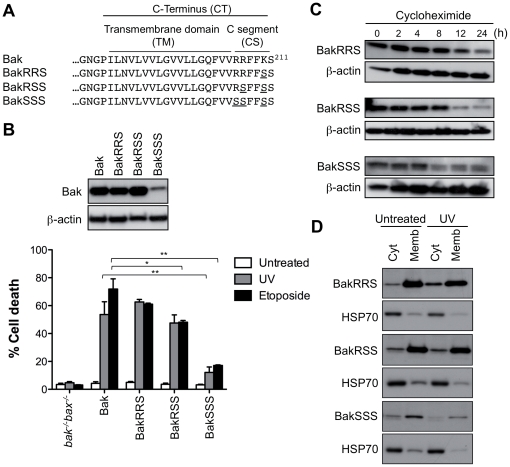
Basic residues in the C-segment are necessary for Bak stability and function. (**A**) C-terminal sequence of Bak variants indicating which basic residues in the C-segment that were substituted with serine. (**B**) Substitution of basic residues in the C-segment reduces Bak proapoptotic function. *bak^−/−^bax^−/−^* MEFs expressing Bak, BakRRS, BakRSS or BakSSS were left untreated, or treated with UV or etoposide for 24 h. Percentage cell death is expressed as the mean ± SEM from three independent experiments. Statistical significance for treatment when compared to Bak; *p<0.05, **p<0.01. Upper panel is a western blot of cell lysates immunoblotted for Bak, and for β-actin as a loading control. (**C**) Substitution of basic residues in the C-segment destabilizes Bak. Cells from (B) were incubated with cycloheximide for up to 24 h and cell lysates immunoblotted for Bak, and for β-actin as a loading control. Note that due to low expression of BakSSS, 4-fold total protein was loaded onto the gels. (**D**) Substitution of basic residues does not prevent targeting to membranes. Cells from (B) were left untreated or treated with UV, separated into cytosolic and membrane fractions, and immunoblotted for Bak and for the cytosolic marker HSP70. Results are representative of two or more independent experiments.

Basic residues in the C-segment of tail-anchored proteins can assist in membrane-integration and in targeting specific membranes [30], [32], [33]. To examine the role of the three basic residues in the Bak C-segment, one, two, or all three, were replaced with serine (Figure 2A). Substitution of all three basic residues (BakSSS) had a similar effect to C-segment truncation, as the protein was unstable as shown by low expression level and low half-life, with cytochrome *c* release and apoptosis greatly decreased (Figures 2B and C and Figure S1). Substitution of the last two basic residues (BakRSS) slightly blocked apoptotic function (Figure 2B and Figure S1) despite high protein levels (Figure 2B) and efficient membrane targeting (Figure 2D). This diminished function of BakRSS may be due to targeting to non-mitochondrial membranes, as reported for Bcl-x_L_ when either basic residue in the C-segment was replaced [39], although attempts to examine this by both cell fractionation and confocal microscopy were not conclusive (data not shown). Finally, substitution of a single basic residue at two positions had little effect on localization or apoptotic function (BakRRS, BakSRK; Figures 2B and D and data not shown). While we have not tested whether the second or third basic residue alone (e.g. BakSRS or BakSSR) may efficiently target Bak to mitochondria, our data suggest that two basic residues in the Bak C-segment are necessary for efficient proapoptotic function, as shown for Bcl-x_L_ [39]. However, a third basic residue may contribute to Bak stability, as BakRRS had a slightly lower half-life compared to Bak, and many vertebrate Bak proteins contain three basic residues in the C-segment.

### Substitution of the Bak C-segment with that of Bax preserves Bak stability and function, but confers cytosolic localization

**Figure 3:**
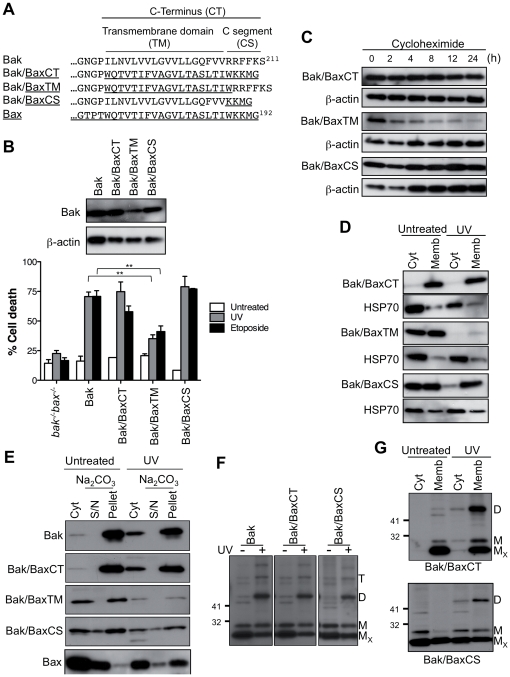
The Bax C-segment converts Bak to a semi-cytosolic protein. (**A**) C-terminal sequence of Bak/Bax chimeras. (**B**) Bak containing the Bax C-terminus or C-segment retains stability and function. *bak^−/−^bax^−/−^*MEFs expressing Bak or Bak/Bax chimeras were left untreated or treated with UV or etoposide. Percentage cell death is expressed as the mean ± SEM from three independent experiments. Statistical significance for treatment when compared to Bak; **p<0.01. Upper panel is a western blot of cell lysates immunoblotted for Bak and for β-actin as a loading control. (**C**) Bak containing the Bax C-terminus or C-segment retains stability. Cells from (B) were incubated with cycloheximide and cell lysates immunoblotted for Bak, and for β-actin as a loading control. (**D**) Bak containing the Bax transmembrane domain or C-segment locates to both cytosolic and membrane fractions. Cells from (B) were left untreated or treated with UV, separated into cytosolic and membrane fractions and immunoblotted for Bak or Bax, and for the cytosolic marker HSP70. (**E**) Bak/Bax chimeras in the membrane fraction are mostly membrane-integrated. Cells were treated as in (D) and the membrane fractions treated with sodium carbonate to extract peripherally attached proteins. Samples were immunoblotted for Bak or Bax. (**F**) Bak/Bax chimeras retain ability to change conformation and oligomerize during apoptosis. Cells were treated as in (D) and the cell lysates exposed to oxidant (CuPhe), run on non-reducing SDS-PAGE, and immunoblotted for Bak. M_X_, non-activated intramolecular crosslinked monomer; M, non-crosslinked monomer; D, intermolecular crosslinked dimers; T, crosslinked trimers. (**G**) Following UV, oligomerized Bak/BaxCS is mainly at mitochondria. Cells were treated as in (D), separated into cytosolic and membrane fractions, run on non-reducing SDS-PAGE and immunoblotted for Bak. Results are representative of two or more independent experiments.

To explore the role of the C-termini in the differential localization of Bak and Bax we generated three chimeras in which the whole Bak C-terminus, or just the TM domain or C-segment was replaced with that of Bax (Figure 3A). Bak containing the Bax C-terminus (Bak/BaxCT) exhibited the characteristics of wild-type Bak in terms of expression level, half-life, mitochondrial localization, oligomerization and proapoptotic function (Figures 3B, C, D, E, F, G and Figure S1). Thus, the Bax C-terminus could act as a fully functional membrane anchor for Bak. The protein was fully membrane-bound prior to apoptosis, presumably due to inability of the Bax TM domain to be sequestered in the Bak groove.

Bak containing the Bax TM domain (Bak/BaxTM) was relatively unstable. The protein displayed reduced proapoptotic function, short half-life, and rapid degradation following UV (Figures 3B, C, D, E and Figure S1). This chimera also exhibited impaired targeting to membranes (Figures 3D and E), suggesting that the secondary structure of the “mixed” C-terminus may not be optimal for membrane insertion. As the membrane-integrated population was also unstable (Figures 3D and E), the Bak C-terminus may not only be important for targeting and insertion, but also for stability of the protein once membrane anchored.

Notably, Bak containing the Bax C-segment (Bak/BaxCS) was fully functional in mediating apoptosis, as indicated by cytochrome *c* release and by loss of cell viability in a caspase-dependent manner (Figure 3B and Figures S1 and S2). Accordingly, the protein exhibited normal half-life (Figure 3C). However, instead of being located solely in the mitochondrial fraction, a significant portion of the protein was now cytosolic in healthy cells (Figures 3D and E). The portion of Bak/BaxCS that did locate to mitochondria was partially inserted as indicated by carbonate resistance (Figures 3E). Following apoptotic signalling, Bak/BaxCS still underwent conformation change and oligomerization following UV (Figures 3F and G) or actinomycin D (Figure S3), with oligomerization predominantly in the membrane fraction. A variant of this chimera that contains five (WKKMG) rather than four (KKMG) Bax residues displayed similar subcellular localization and proapoptotic function (Bak/BaxCS^b^; Figure S4 and data not shown). In summary, Bak/BaxCS remained functional despite significant initial cytosolic localization.

**Figure 4:**
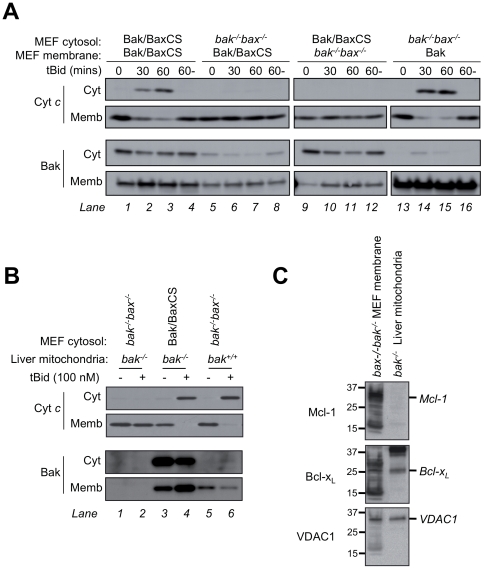
Bak/BaxCS translocates to mitochondria and following tBid releases cytochrome *c*. (**A**) Cytosolic and mitochondrial Bak/BaxCS both contribute to permeabilization of MEF mitochondria. Cytosol and membrane fractions from *bak^−/−^bax^−/−^* MEFs or from *bak^−/−^bax^−/−^* MEFs expressing either Bak or Bak/BaxCS were combined as shown, and incubated at 30°C in the presence of 100 nM tBid for 0, 30 or 60 mins, or without tBid for 60 mins (60-). Supernatant (*Cyt*) and membrane (*Memb*) fractions were immunoblotted for cytochrome *c* and for Bak. (**B**) Cytosolic Bak/BaxCS is sufficient to permeabilize mouse liver mitochondria. MEF cytosol fractions derived as in (A) were combined with mitochondria isolated from wild-type (*bak^+/+^*) or *bak^−/−^* mouse liver, and incubated at 37°C with or without 100 nM tBid for 60 min. Supernatant (*Cyt*) and membrane (*Memb*) fractions were immunoblotted for cytochrome *c* and for Bak. Note that Bak/BaxCS levels appear high compared to the endogenous mouse Bak, possibly due to different recognition by the anti-Bak antibody. (**C**) Mcl-1 is higher in MEF membranes than in mouse liver mitochondria. MEF membranes and mouse liver mitochondria at the concentrations used in (A) and (B) were examined for levels of Mcl-1, Bcl-x_L_ and VDAC1 by immunoblotting. Results are representative of two or more independent experiments.

To examine in more detail whether the cytosolic fraction of Bak/BaxCS can translocate and permeabilize mitochondria, a series of experiments were performed. Subcellular fractionation showed that after treatment with apoptotic stimlui, Bak/BaxCS is predominantly at mitochondria (Figures 3D, E and G and Figure S3), although partial loss of Bak/BaxCS following UV treatment precluded clear conclusions regarding translocation (e.g. Figures 3D and E). By confocal microscopy, Bak/BaxCS (tagged with FLAG at the N-terminus) also increased at mitochondria in a portion of cells after treatment with etoposide (Figure S5), although translocation was not marked due to the portion of Bak/BaxCS already resident at mitochondria. We then tested translocation and permeabilization in cell-free assays by combining Bak/BaxCS cytosolic extracts with mitochondria from two different sources (Figure 4). When MEF cytosol containing Bak/BaxCS was “recombined” with MEF membranes containing Bak/BaxCS, the addition of tBid triggered cytochrome *c* release (Figure 4A, lanes 2 and 3). However, cytochrome *c* was not released if either fraction was combined with cytosol or membranes from *bak^−/−^bax^−/−^* MEF (Figure 4A, lanes 6,7, 10 and 11), indicating that both the cytosolic and mitochondrial portions of Bak/BaxCS are required to reach the threshold of Bak necessary for cytochrome *c* release in this assay. Blotting for Bak indicated that this threshold (lanes 2 and 3) includes resident mitochondrial Bak/BaxCS (e.g. lane 5) as well as cytosolic Bak/BaxCS recruited to mitochondria in the absence of tBid (e.g. lane 9) and in the presence of tBid (e.g. lane 10).

In a second cell-free assay (Figure 4B), MEF cytosol fractions were incubated with mouse liver mitochondria which contain very low levels of Bcl-x_L_ and Mcl-1 [41]. Cytosolic Bak/BaxCS was now able to mediate complete cytochrome *c* release by tBid (Figure 4B, lane 4). Again, a portion of Bak/BaxCS was recruited to mitochondria in the absence of tBid, with further protein recruited during tBid incubation (lanes 3 and 4). While the recruited Bak/BaxCS (lanes 3 and 4) appears greater than the resident mouse Bak in *bak^+/+^* liver mitochondria (lanes 5 and 6), the anti-Bak antibody was raised against a peptide derived from the human 23–38 sequence that differs from that of mouse Bak. Notably, in this assay the cytosolic Bak/BaxCS protein was solely responsible for cytochrome *c* release as liver mitochondria from *bak^−/−^* mice are essentially devoid of Bak and Bax [41]. A direct comparison of Mcl-1 levels in the two sources of mitochondria (Figure 4C) suggests that a low Mcl-1 level in mouse liver mitochondria confers a low threshold of Bak required for cytochrome *c* release. In summary, in these cell-free assays, cytosolic Bak/BaxCS could translocate to the mitochondrial fraction, and contribute to cytochrome *c* release initiated by tBid (Figure 4). These findings, together with evidence of translocation following apoptotic signalling in cells (Figure 3 and Figures S3 and S5), indicate that in cells undergoing apoptosis (Figure 3), both the cytosolic and mitochondrial fractions of Bak/BaxCS are functional.

### A TM: Groove interaction contributes to the cytosolic localization of Bax and Bak/BaxCS

**Figure 5:**
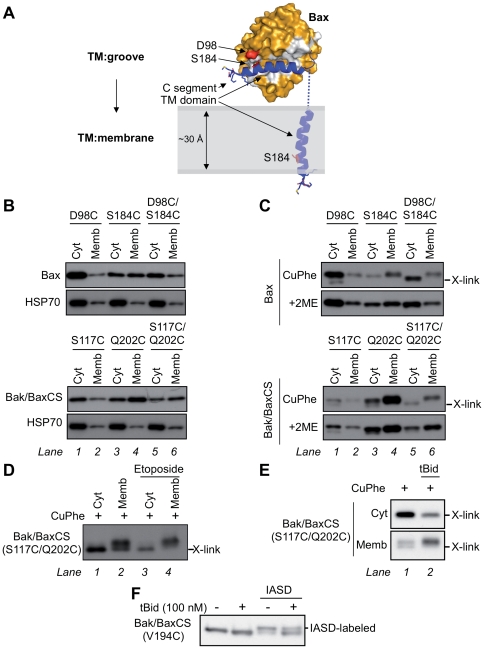
A TM:groove interaction forms in cytosolic Bax and in cytosolic Bak/BaxCS. (**A**) Schematic of Bax converting from a TM:groove to a TM:membrane conformation during apoptosis. Bax is shown as surface representation (*orange*) with the hydrophobic surface groove highlighted (*white*). The Bax TM domain is represented (*blue*) and the C-segment (KKMG) has the sidechains shown. Residues proposed to interact during TM:groove interaction are highlighted (S184 and D98; *red*). Images were generated in Pymol using the RCSB Protein Data Bank file 1F16 for Bax [38]. (**B**) Mutations in both the TM domain and the groove alter mitochondrial targeting. Cytosolic and membrane fractions from *bak^−/−^bax^−/−^* MEFs expressing the indicated Bax or Bak/BaxCS cysteine mutants were immunoblotted for Bax, Bak/BaxCS, or HSP70. (**C**) A TM:groove interaction forms in cytosolic Bax and in cytosolic Bak/BaxCS. Cytosolic and membrane fractions from cells in (B) were incubated with oxidant (CuPhe) and electrophoresed under non-reducing conditions (*upper*) or reducing conditions (+2ME, *lower*) and immunoblotted for Bax or Bak/BaxCS. Intramolecular cysteine linkage (D98C:S184C in Bax; S117C:Q202C in Bak/BaxCS) results in faster migration under non-reducing conditions (*X-link*). (**D**) Etoposide treatment decreases TM:groove interaction in membrane-associated Bak/BaxCS. Cells expressing the Bak/BaxCS S117C/Q202C variant were incubated with or without etoposide for 24 h. Cytosolic and membrane fractions were incubated with CuPhe, electrophoresed under non-reducing conditions, and immunoblotted for Bak. (**E**) tBid treatment decreases the TM:groove interaction in membrane-associated Bak/BaxCS. Cells in (D) were permeabilized and incubated with or without tBid (100 nM). Cytosolic and membrane fractions were separated and then incubated with CuPhe, electrophoresed under non-reducing conditions, and immunoblotted for Bak. (**F**) The TM domain of Bak/BaxCS inserts into membranes following tBid. Permeabilized cells were treated with or without tBid as in (E), then incubated with or without IASD. Membrane fractions were electrophoresed under reducing conditions and immunoblotted for Bak. Results are representative of two or more independent experiments.

As discussed above, structure and mutagenesis studies found that cytosolic Bax is stabilized by a TM:groove interaction possibly involving hydrogen bonding between S184 (TM) and D98 (groove) (Figure 5A) [42], [43]. Based on these findings, we used disulphide linkage to examine whether the cytosolic populations of Bax and Bak/BaxCS adopt a TM:groove interaction. We first substituted the two endogenous cysteines in Bax (C62 and C126) and in Bak/BaxCS (C14 and C166) for serine. Then cysteine residues were placed in the TM and groove at positions predicted to interact in Bax (D98C/S184C), and at equivalent positions in Bak/BaxCS (S117C/Q202C). As controls, single cysteine substitutions in Bax (D98C and S184C) and Bak/BaxCS (S117C and Q202C) were also examined. Each Bax and Bak/BaxCS cysteine variant retained proapoptotic function when stably expressed in *bak^−/−^bax^−/−^*MEFs (Figure S6 and unpublished data).

We first noted that subcellular localization was altered by the cysteine substitutions, as cysteine in the TM of both Bax (S184C) and Bak/BaxCS (Q202C) increased localization to the membrane fraction (Figure 5B, lanes 4). As cysteine is more hydrophobic than serine or glutamine, it may encourage membrane insertion, as previously reported for Bax in which S184 was substituted with a more hydrophobic residue (BaxS184V, S184A and S184L) [30], [44].

To then test whether cysteines in the TM and groove were in close proximity in cytosolic Bax and Bak/BaxCS, we tested whether the two cysteines could form an intramolecular disulphide bond after addition of the oxidant, copper (I,II) phenanthroline (CuPhe). As predicted by the Bax structure, intramolecular cysteine linkage was apparent for the double-cysteine Bax variant (D98C/S184C) in the cytosolic fraction, as under non-reducing conditions (CuPhe) the protein migrated further than the single-cysteine variants (Figure 5C, compare lane 5 to lanes 1–4). Cysteine linkage was also apparent for the double-cysteine variant of Bak/BaxCS (S117C/Q202C) compared to the single-cysteine variants (Figure 5C, compare lane 5 with lanes 1–4). Disulphide linkage was responsible for the fast migration as the reducing agent 2-mercaptoethanol (2ME) prevented faster migration for both double-cysteine variants (Figure 5C, lower gels, lanes 5). Cysteine linkage was not evident in the membrane fractions of the double-cysteine variants, likely due to membrane insertion of the TM domain (Figure 5C, lanes 6). In summary, a TM:groove interaction was evident both in cytosolic Bax and in cytosolic Bak/BaxCS.

We next examined whether Bak/BaxCS and Bax convert from a TM:groove conformation to a TM:membrane conformation during apoptosis, as depicted for Bax in Figure 5A. Cells expressing the double-cysteine Bak/BaxCS variant (S117C/Q202C) were treated with etoposide to induce apoptosis, and the cell fractions treated with CuPhe and examined for TM:groove linkage (X-link; Figure 5D). Here, TM:groove linkage was evident not only in the cytosolic fraction but in part of the membrane-associated protein prior to etoposide, presumably due to some Bak/BaxCS being peripherally attached to mitochondria, as indicated by carbonate extraction (Figure 3E) and by partial dissociation from membranes during a 30 min incubation in *bak^−/−^bax^−/−^* MEF cytosol (Figure 4A, lanes 5–8). Notably however, TM:groove linkage in the membrane fraction decreased after etoposide (Figure 5D, compare lanes 2 and 4). (Unfortunately, several experiments with the double-cysteine Bax variant could not clearly distinguish a decrease in TM:groove linkage.) In a second approach, cell extracts from these cells were incubated with tBid (as in Figure 4A), and cytosol and membrane fractions subjected to oxidation and examined for TM:groove linkage (X-link; Figure 5E). tBid caused the double-cysteine Bak/BaxCS variant to translocate to the membrane fraction, and decreased the proportion of protein that could undergo TM:groove linkage (Figure 5E). The observed decrease in the TM:groove conformation of Bak/BaxCS after either etoposide or tBid is consistent with insertion of the TM domain (that contains the Cys202 residue) into the mitochondrial OM.

To more directly monitor membrane insertion of the Bak/BaxCS TM domain, a cysteine labelling approach was used (Figure 5F). Cysteine can be labelled irreversibly by 4-acetamido-4′-((iodoacetyl) amino) stilbene-2,2′-disulfonic acid (IASD), except if the cysteine is positioned in hydrophobic environments such as lipid bilayers [45]. Cysteine was thus introduced at position V194 in the TM domain of Bak/BaxCS, and the protein found to retain proapoptotic function (data not shown). Cell lysates were then incubated with or without tBid and subjected to IASD-labelling. Bak/BakCS in membrane fractions showed an increase in molecular weight conferred by the IASD molecule (624 Da). While most of the membrane-associated V194C could be labelled before tBid, labelling decreased after tBid (Figure 5F). Thus, cysteine linkage and cysteine labelling both indicate that Bak/BaxCS translocation to mitochondria during apoptosis involves conversion from a TM:groove to a TM:membrane conformation, as depicted for Bax in Figure 5A.

### Regulation of Bak and Bax is independent of initial subcellular localization

**Figure 6:**
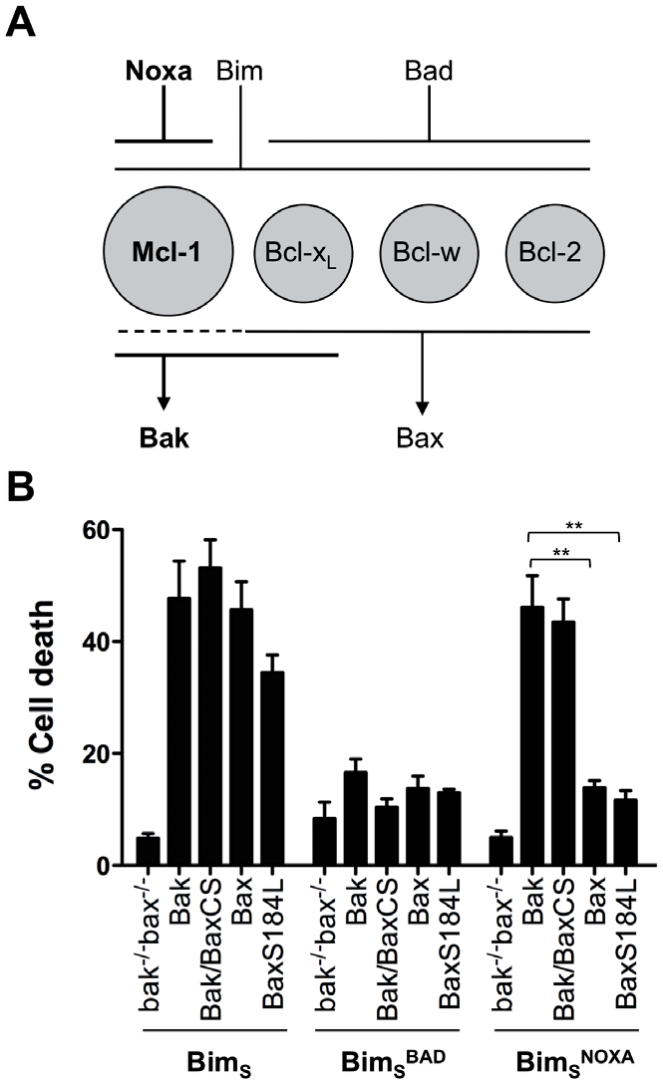
Bak regulation is independent of initial subcellular localization. (**A**) Schematic of Bak-mediated apoptosis initiated by Noxa-Mcl-1 signalling in MEFs. The four prosurvival proteins expressed in MEF (Mcl-1, Bcl-x_L_, Bcl-w and Bcl-2) are depicted, together with their preferential binding of BH3-only proteins (Noxa, Bim and Bad) and of Bak and Bax [20], [46]. The Noxa-Mcl-1-Bak pathway to apoptosis is indicated (*bold*). (**B**) Expression of Bim_S_^NOXA^ preferentially mediates apoptosis via either Bak or Bak/BaxCS. *bak^−/−^bax^−/−^*MEFs expressing the indicated Bak and Bax variants were retrovirally infected with Bim_S_ or with Bim_S_ containing the Bad or Noxa BH3 domains (Bim_S_^BAD^ and Bim_S_^NOXA^). Percentage cell death at 36 h (normalized to the efficiency of infection) is expressed as the mean ± SEM of three independent experiments. Statistical significance for treatment when compared to Bak; **p<0.01.

How Bak and Bax are regulated by prosurvival Bcl-2 proteins is a major enigma in the field. Having shown that Bak/BaxCS is semi-cytosolic and that the cytosolic portion of Bak/BaxCS was important for cell death in these cells, we examined whether subcellular localization contributes to the differential regulation of Bak and Bax. For example, do apoptotic signals that preferentially initiate Bak-dependent apoptosis, also initiate Bak/BaxCS-dependent apoptosis? To test this we used a retroviral approach to express three Bim_S_ variants in MEFs that already stably express different Bak and Bax variants. The three Bim_S_ variants were wild type Bim_S_, or Bim_S_ in which the BH3 domain was swapped with the BH3 domain of Bad (Bim_S_^BAD^) or of Noxa (Bim_S_^NOXA^). The predicted effects of each Bim_S_ variant is described in the schematic in Figure 6A, which depicts Mcl-1 as the principal guardian of Bak in MEFs (see Figure 4C), and describes the binding profiles of Noxa, Bim and Bad to prosurvival proteins, and the binding profiles of prosurvival proteins to Bak and Bax [18], [19], [20], [46].

We used *bak^−/−^bax^−/−^*MEFs that had been stably transfected with Bak, Bak/BaxCS, Bax or a mitochondria-targeted BaxS184L variant [44]. In each of these cells lines, expression of Bim_S_ initiated apoptosis (Figure 6B), explained by strong binding of Bim_S_ to all prosurvival proteins (and perhaps direct activation of both Bak and Bax by Bim_S_). In contrast, expression of Bim_S_^BAD^ failed to induce apoptosis in any cell line, explained by weak binding of Bad to Mcl-1. Notably, Bim_S_^NOXA^ efficiently initiated apoptosis in cells expressing Bak because Noxa binds strongly to Mcl-1 to leave Bak unguarded. In contrast, Bim_S_^NOXA^ did not kill cells expressing Bax because Noxa does not bind to three Bax guardians (Bcl-2, Bcl-w and Bcl-x_L_). Thus, this approach distinguishes between Bak- and Bax-mediated apoptosis. We then noted that Bim_S_^NOXA^ was still able to initiate cell death via Bak/BaxCS as efficiently as via wild-type Bak, indicating that the semi-cytosolic locale of Bak/BaxCS did not alter its regulation.

We note that in these experiments, Noxa expression alone was sufficient to kill *bak^−/−^bax^−/−^* MEFs expressing human Bak, presumably because Mcl-1 is the principal guardian of Bak in these cells and because Noxa can directly activate Bak [47]. This contrasts with earlier studies in which Noxa required co-expression of Bad to kill *bax^−/−^*MEFs [46]. One possibility is that in the present studies, enforced expression of hBak has altered the profile of prosurvival proteins to make Mcl-1 dominant. Consistent with this, altering the prosurvival profile by overexpressing Bcl-x_L_ made the cells resistant to Noxa-induced killing (Figure S7).

Bak and Bax regulation in these cells was also tested by initiating apoptosis with UV, cycloheximide and actinomycin D, three agents that are thought to initiate Bak-dependent apoptosis by degrading Mcl-1 and/or by increasing Noxa expression [20], [26], [48]. Accordingly, these stimuli preferentially triggered apoptosis via Bak or Bak/BaxCS, whereas etoposide induced apoptosis equally via Bak, Bak/BaxCS or Bax (Figure S8). The major role of Mcl-1 in regulating Bak and Bak/BaxCS in these cells is supported by experiments in which both Bak and Bak/BaxCS readily co-precipitated with Mcl-1, while Bax and BaxS184L did so only poorly (Figure S9). Thus, Bak-mediated apoptosis initiated by targeting Mcl-1 is independent of the initial intracellular localization of Bak.

## Discussion

### The Bak transmembrane domain and C-segment both contribute to mitochondrial targeting and membrane insertion

The C-termini of tail-anchored proteins, including Bak and Bax, contain an α-helical transmembrane domain often flanked by basic residues [32], [33]. Accordingly, in Bak, truncation of the whole C-terminus (25 residues) rendered Bak cytosolic and non-functional when stably expressed in *bak^−/−^bax^−/−^* MEFs (Figure 1) [29], as reported for transient transfection of HeLa cells [28]. Likewise, truncation of the Bax C-terminal 23 residues also blocked its function in *bak^−/−^bax^−/−^*MEFs (data not shown), as recently shown for Bax with GFP fused to the C-terminus [31]. Truncation of just the C-segment also rendered Bak largely cytosolic and non-functional, as reported for truncation of the C-segment of Bax [30]. Substitution of the three basic residues in the Bak C-segment also significantly decreased half-life and function. Thus, our results based on stable expression of Bak and Bax in Bak/Bax-deficient cells verify that the C-terminus of each protein is necessary for targeting and insertion into the mitochondrial outer membrane, and show that the C-segment within these regions plays a vital role. Somewhat curiously, the C-terminus, or C-terminus-dependent mitochondrial localization, was also needed for Bak activation and conformation change, as truncation of the C-terminus essentially prevented Bak conformation change in response to tBid or UV (Figure 1E) [8].

### Bak/BaxCS, a semi-cytosolic functional form of Bak

A semi-cytosolic form of Bak, Bak/BaxCS, was generated by replacing the Bak C-segment (RRFFKS) with that of Bax (KKMG). This chimera retained proapoptotic function despite its significant distribution in the cytosol, suggesting that it may translocate like Bax to mitochondria and permeabilize the OM. Indeed, translocation of the cytosolic portion to mitochondria was observed in cell-free assays and in cells. Notably, recruitment of cytosolic Bak/Bax to MEF mitochondria was necessary to reach the threshold of mitochondrial Bak needed for OM permeabilization in cell-free assays, suggesting that Bak/BaxCS translocation is also necessary for OM permeabilization in intact MEF cells. The ability of cytosolic Bak/BaxCS and of cytosolic Bax to translocate to mitochondria, oligomerize and permeabilize the OM during apoptosis suggests a conserved mode of activation for Bak and Bax.

In the cytosol, Bak/BaxCS was able to sequester the hydrophobic TM domain in the hydrophobic surface groove, as shown by cysteine linkage. A similar TM:groove interaction in cytosolic Bax was also evident, as proposed by structural and mutagenesis studies [42], [43]. A TM:groove interaction was less evident in mitochondrial Bak/BaxCS, especially following apoptotic signalling, indicating that the TM domain moves from the hydrophobic groove to insert into the hydrophobic OM. Actual insertion into the OM following tBid was indicated by decreased labelling of cysteine introduced into the TM domain of Bak/BaxCS.

A TM:groove interaction in cytosolic Bak/BaxCS also implies that although the Bak groove appears “closed” in the structure of non-activated Bak [49], it may “open” to accommodate the TM domain while remaining non-activated. Thus, plasticity evident in the hydrophobic grooves of the prosurvival proteins Bcl-x_L_ and A1 [50] may extend to the hydrophobic groove in Bak. Indeed, a recent study showed that the Bak groove could transiently bind activator BH3-only proteins, initiating Bak activation [47]. A much greater change in the groove is presumably involved in Bak activation, where the groove opens to expose the BH3 domain and then bind the BH3 domain of another activated Bak molecule, resulting in symmetric dimers [8], [29].

How the Bax C-segment in Bak/BaxCS promotes a TM:groove interaction is not clear. It is possible that the Bax C-segment (KKMG) forms opportunistic interactions with negatively charged residues on the groove surface (e.g. E105 and E109 in Bak α-helix 4), which then stabilize TM binding to the body of the protein. Alternatively, the hybrid of Bak TM domain with Bax C-segment may hinder membrane insertion, and so skew Bak/BaxCS towards a TM:groove interaction in the cytosol. It is unlikely that the Bax C-segment acts by altering Bak/BaxCS binding to proteins in the cytosol or mitochondria, as cells sorted for low or high levels of Bak/BaxCS exhibited a similar distribution of cytosolic *vs* mitochondrial Bak/BaxCS (data not shown).

### The role of the C-terminus in Bak stability and conformation

Certain changes to the Bak C-termini decreased protein stability or altered conformation, highlighting the importance of this region in Bak function. Bak half-life was decreased by truncating the C-segment (but not the whole C-terminus) presumably because an exposed hydrophobic TM domain targets Bak for degradation. In contrast, Bax lacking the C-segment was stable (data not shown), perhaps due to a strong TM:groove interaction. TM-driven degradation is not likely to contribute to turnover of wild-type Bak, as the TM domain is normally membrane-inserted. In relation to Bak conformation, truncating the whole C-terminus (BakΔCT) encouraged unfolding of the N-terminus, as a portion of the protein failed to form a C14:C166 crosslink (Figure 1E) [8], and exposure of N-terminal epitopes could be detected (data not shown). Others have reported N-terminal epitope exposure in Bax after truncation of the C-terminal 21 residues [30], although this effect must be indirect as the N- and C-termini are not in contact in the Bax structure [38].

### Bak regulation is independent of mitochondrial localization

Conversion of Bak to a semi-cytosolic protein (i.e. Bak/BaxCS) did not alter its regulation by other Bcl-2 proteins. We were able to test this in cells where Noxa-Mcl-1 signalling preferentially initiates Bak-mediated rather than Bax-mediated apoptosis. In that system, Bak/BaxCS responded like Bak rather than like Bax. In addition, a mitochondrial Bax protein (S184L) responded like Bax rather than like Bak. Thus, differences in the regulation of Bak and Bax [24], [25] are best explained by specific binding of Bak and Bax with other Bcl-2 proteins such as Mcl-1, with this binding being largely unaffected by initial subcellular localization.

### Conclusions

The C-termini are important for Bak and Bax stability in the cytosol and mitochondrial OM, for their translocation and insertion into the OM, and for their conformation change and oligomerization to finally form the apoptotic pore. Central to these functions is conversion from a TM:groove to a TM:membrane conformation either before apoptosis (Bak), or during apoptosis (Bax, Bak/BaxCS). The conversion is dependent not only by residues within the TM domain and the groove, but by residues within the C-segment. Further studies are needed to confirm whether, during apoptosis, converting from a TM:groove to a TM:membrane conformation is triggered by TM dislodgement from the groove by BH3-only proteins, post-translational modification, or changes to the intracellular environment [7], [27], [38], [47], [51].

## Materials and Methods

### Ethics Statement

We used approximately 20 week old wild type or *bak^−/−^* C57BL-6 mice for this study. All animal care and experimental procedures were conducted according to the guidelines of the Australian Code of Practive for the Care and Use of Animals for Scientific Purposes, 7^th^ Edition 2004, and the WEHI Animal Ethics Committee, and were approved by the Royal Melbourne Hospital Animal Ethics Committee (Approval #2009.012).

### Cell culture, retroviral infection, induction of cell death, apoptosis assays

Mouse embryonic fibroblasts (MEFs) derived from *bak^−/−^bax^−/−^* mice were generated and transformed with SV40 large T as described [23]. MEFs were maintained in Dulbecco's Modified Eagles medium supplemented with 10% fetal calf serum, 250 mM L-asparagine and 55 mM 2-mercaptoethanol.

Variants of human Bak and Bax were generated by site-directed mutagenesis (primer sequences available on request). Variants were then stably expressed in *bak^−/−^bax^−/−^* MEFs by retroviral infection of pMX-IG (IRES-GFP) retroviral constructs that were first introduced into Phoenix cells by FuGENE 6-mediated transfection according to the manufacturer's protocol (Roche). Viral supernatants were then used to infect MEFs as previously described [46]. Polyclonal populations of Bak- and Bax-expressing cells (GFP-positive) were selected by FACSorting. For stable expression of FLAG-Bak, FLAG-Bak/BaxCS or FLAG-Bcl-x_L_, constructs were cloned into a pMIH (IRES-Hygro) retroviral vector and polyclonal populations of infected cells selected by serial passage in hygromycin.

To induce apoptosis, cells were treated with apoptotic stimuli (100 J/m^2^ UV; 1 µM actinomycin D (ActD); 10 µM etoposide; 50 µg/ml cycloheximide) and incubated at 37°C for 24 h, unless indicated otherwise. Where indicated, cells were also co-incubated in the presence of the broad range caspase inhibitor Q-VD.oph (50 µM, Enzyme Systems, CA). To measure cell death, floating cells were combined with trypsin-detached cells, resuspended in phosphate-buffered saline containing 5 µg/ml propidium iodide, and incubated on ice for 5 min before measuring the percentage non-viable cells (propidium iodide positive) by flow cytometry. Where indicated, the cell death data was analyzed by two-tailed Student's t-test.

### Bak stability after protein synthesis inhibition

To estimate protein half-life, *bak^−/−^bax^−/−^* cells expressing Bak and Bax mutants were treated with cycloheximide (50 µg/ml). At the indicated time points cells were harvested, and cell lysate immunoblotted for Bak (Cat #B5929, Sigma), Bax (N20, Santa Cruz Biotechnologies), or for β-actin (Sigma) as a loading control.

### Bak subcellular localization and membrane insertion

To assess the subcellular localization of each Bak variant, MEFs were harvested, washed in ice-cold PBS, and the cell membrane permeabilized by resuspending cells at 1×10^7^ cells ml^−1^ in permeabilization buffer (20 mM HEPES/KOH pH 7.5, 100 mM sucrose, 2.5 mM MgCl_2_, 100 mM KCl, 1 mM DTT, 0.025% digitonin, Complete protease inhibitors (Roche), and in most cases 4 µg/ml pepstatin A (Sigma)). After incubation on ice for 10 min, cell membrane permeabilization was verified by uptake of trypan blue, the cells centrifuged at 13,000 *g* for 5 min and equivalent amounts of cytosol (Cyt) and membrane (Memb) fractions immunoblotted for Bak, HSP70 as a cytosolic marker (HSP70 antibody is a gift from Dr W. Welch and Dr R. Anderson).

To assess membrane insertion of Bak, membrane fractions were resuspended in 0.1 M Na_2_CO_3_ (pH 11.5) and incubated on ice for 20 min. pH was neutralized with 0.1 M HCl and incubated for 5 min before addition of 10× nuclease buffer (400 mM Tris HCl, 100 mM MgSO_4_, 10 mM CaCl_2_) and 1 unit of DNAase I (Promega), and incubation at 37°C for 10 min. Samples were centrifuged at 13,000 *g* for 10 min and supernatant and pellet fractions immunoblotted for Bak.

### Detection of Bak conformation by disulphide bond formation

MEFs were permeabilized by 0.025% digitonin in cross-linking buffer (20 mM HEPES/KOH pH 7.5, 100 mM sucrose, 2.5 mM MgCl_2_, 50 mM KCl), and membranes resuspended in the same buffer without digitonin. For disulphide bond formation, cytosol and membrane fractions were incubated with the redox catalyst copper(II)(1,10-phenanthroline)_3_ (CuPhe) on ice for 30 min. The CuPhe stock was 30 mM CuSO_4_ and 100 mM 1,10-phenanthroline in 4∶1 water/ethanol and diluted 100-fold into the sample. Oxidizing conditions were quenched by 20 mM EDTA to chelate copper and 20 mM N-ethylmaleimide to block unreacted cysteine residues, and the samples analyzed by non-reducing SDS-PAGE.

### Isolation of mouse liver mitochondria

Mouse liver mitochondria (MLM) were isolated from approximately 20-week old C57Bl-6 wild type or *bak^−/−^* mice as previously described [41]. MLM were resuspended at 0.5 mg/ml in MEF cytosolic extracts and incubated with or without caspase-8-cleaved Bid (tBid) for 60 min at 37°C.

### Mitochondrial outer membrane permeabilization by tBid

Permeabilized cells, or combinations of membrane and cytosol fractions generated as above, were incubated with tBid for 30 or 60 min at 30°C. Where indicated, cytosol derived from MEFs was incubated with MLM and incubated with tBid as above. Supernatant and membrane fractions were separated by centrifugation (13,000 *g* for 5 min) and fractions transferred to nitrocellulose for immunoblotting for cytochrome *c*, or to PVDF for immunoblotting for Bak.

### IASD labelling

Cytosol and membrane fractions were separated and treated with 1 mM IASD (4-acetamido-4′-((iodoacetyl) amino) stilbene-2,2′-disulfonic acid, Invitrogen) in the presence of 0.1 mM TCEP (Tris(2-Carboxyethyl)phosphine; Pierce) for 30 mins at room temperature. Labelling was quenched with 1 mM dithiothreitol and labelling assessed by SDS-PAGE and immunoblotting for Bak.

### Preferential Bak activation by Noxa overexpression

*bak^−/−^bax^−/−^* MEFs expressing Bak, Bax, Bak/BaxCS or Bax S184L were retrovirally infected with Bim_S_ or Bim_S_ containing the BH3 domain from Bad or Noxa (provided by D.C.S. Huang, and described in [46]). After 36 hours, cell death was assessed by propidium iodide uptake. Percentage cell death was calculated by normalizing against the rate of infection (GFP-positive cells) of the three Bim_S_ constructs for *bak^−/−^bax^−/−^* cells.

## Supporting Information

Figure S1 **Mitochondrial cytochrome** ***c*** **release mediated by Bak variants.** *bak^−/−^bax^−/−^* MEFs expressing the indicated Bak variants were left untreated (*black line*) or treated with UV (*red lines*) as in Figures 1B, 2B and 3B and examined for mitochondrial cytochrome *c (Cyt c)* content by flow cytometry (Waterhouse and Trapani, *Cell Death Differ* 2003 Jul; **10** (7): 853–855) using anti-cytochrome *c* antibody (6H2.B4; Pharmingen) and R-phycoerythrin-conjugated secondary antibody (1031-09; Southern Biotech, AL, USA). Untreated cells were incubated with secondary antibody only (*hatched*). Cells with low mitochondrial cytochrome *c* content lie to the left of the marker line. Results are representative of two independent experiments.
(TIF)

Figure S2 **Cell death mediated by Bak/BaxCS is apoptotic.** (**A**) Cell death mediated by Bak/BaxCS is blocked by caspase inhibition. *bak^−/−^bax^−/−^* MEFs expressing either wild-type Bak or Bak/BaxCS were treated or not with etoposide (10 µM) in the presence or absence of caspase inhibitor Q-VD.oph (50 µM) for 24 h prior to assessment of cell death by propidium iodide uptake. Results are expressed as mean +/− SEM of three independent experiments. (**B**) Cell death mediated by Bak/BaxCS involves cytochrome *c* release. *bak^−/−^bax^−/−^* expressing Bak/BaxCS treated as in (A) were harvested, and cytosolic (*Cyt*) and membrane (*Memb*) fractions immunoblotted for cytochrome *c*, or for VDAC1 (31HL; Calbiochem) as a mitochondrial membrane marker. Results are representative of two independent experiments.
(TIF)

Figure S3 **Bak/BaxCS oligomerizes predominantly in the membrane fraction during apoptosis.** *bak^−/−^bax^−/−^* MEFs expressing Bak/BaxCS were treated with actinomycin D (1 µM) in the presence of caspase-inhibitor Q-VD.oph (50 µM) for 24 h. Cells were fractionated into cytosol (*Cyt*) and membrane (*Memb*) fractions prior to addition of oxidant (CuPhe) to induce disulphide bonds. Samples were electrophoresed under non-reducing (*upper*) or reducing (*bottom*) conditions prior to immunoblotting for Bak and for HSP70 as a cytosolic marker. M_X_, non-activated intramolecular cross-linked monomer; M, non-crosslinked monomer; D, intermolecular crosslinked dimers. Results are representative of two independent experiments.
(TIF)

Figure S4 **A variant of Bak/BaxCS containing an extra tryptophan residue also retains full pro-apoptotic function.** (**A**) C-terminal sequence of Bak and Bax variants, in particular demonstrating the two Bak/BaxCS variants containing either four (KKMG) or five (WKKMG) residues from Bax. (**B**) Both Bak/BaxCS variants retain proapoptotic function. *bak^−/−^bax^−/−^* MEFs expressing Bak, Bak/BaxCS or Bak/BaxCS^b^ were left untreated or treated with increasing doses of etoposide. Percentage cell death is expressed as the mean ± SEM from three independent experiments. Statistical significance for the 10 mM dose when compared to Bak is shown; *p<0.05. Panels on the right are cell lysates immunoblotted for Bak, and for HSP70 as a loading control.
(TIF)

Figure S5 **Bak/BaxCS redistributes to mitochondria during apoptosis.** (**A**) FLAG-tagged Bak/BaxCS is predominantly cytosolic. Cytosol (*Cyt*) and membrane (*Memb*) fractions were generated from *bak^−/−^bax^−/−^* MEFs stably expressing FLAG-Bak or FLAG-Bak/BaxCS and immunoblotted for FLAG. * is a non-specific band. (**B**) FLAG-Bak/BaxCS translocates to mitochondria following etoposide treatment. FLAG-Bak or FLAG-Bak/BaxCS expressing cells were left untreated or treated with etoposide (10 µM) in the presence of Q-VD.oph (50 µM) for 24 h, and fixed in 4% paraformaldehyde for 20 mins at room temperature. Following staining with an anti-FLAG primary antibody (M2; Sigma) and FITC-conjugated secondary antibody (Southern Biotech, AL, USA), immunofluorescence was detected by confocal microscopy (Leica SP2). All images were obtained using the same parameter settings. Bar represents 20 µm. Note that staining with secondary antibody alone gave essentially no signal (not shown). Images were from a single experiment, representative of two experiments. (**C**) FLAG-Bak/BaxCS colocalizes with mitochondria. Cells were treated as in (B), except that cells were incubated with with MitoTracker Deep Red FM (0.5 µM; Invitrogen, CA) for 30 mins at 37°C prior to fixation and immunostaining. Bar represents 20 µm. Images were from a single experiment, representative of two experiments.
(TIF)

Figure S6 **Cysteine variants of Bax and of Bak/BaxCS retain proapoptotic function.** *bak^−/−^bax^−/−^* MEFs expressing the indicated cysteine variants were left untreated or treated with etoposide. Percentage cell death is expressed as the mean ± SEM from three independent experiments. Statistical significance for the 10 mM dose when compared to wild-type (wt) protein is shown; *p<0.05. Panels on the right are cell lysates immunoblotted for Bax or Bak, and re-blotted for HSP70 as a loading control.
(TIF)

Figure S7 **Noxa-initiated death via Bak/BaxCS is inhibited by Bcl-x_L_.** (**A**) FLAG-Bcl-x_L_ was stably expressed in *bak^−/−^bax^−/−^* MEFs expressing Bak/BaxCS. Cell lysates were immunblotted for FLAG or Bak. (**B**) Bcl-x**_L_** is particularly efficient at blocking Noxa-induced death. *bak^−/−^bax^−/−^* MEFs expressing Bak/BaxCS with or without FLAG-Bcl-x_L_ were retrovirally infected with BimS, or with BimS containing the Noxa or Bad BH3 domains. Percentage cell death was assessed after 36 h by propidium iodide uptake. Data is mean +/− SEM of two experiments. Statistical significance for the effect of Bcl-x_L_ is shown; *p<0.05.
(TIF)

Figure S8 **Bak/BaxCS is regulated like Bak rather than like Bax.** *bak^−/−^bax^−/−^* MEFs stably expressing Bak, Bak/BaxCS or Bax (as in Figure 6) were treated with etoposide (10 mM), UV (100 J/m^2^), cycloheximide (CHX, 50 mg/ml) or actinomycin D (ActD, 1 mM) for 24 h. Percentage cell death is expressed as the mean ± SEM from two independent experiments. Statistical significance for each variant compared to Bak is shown; *p<0.05.
(TIF)

Figure S9 **Mcl-1 binds both Bak and Bak/BaxCS strongly compared to its binding of Bax and BaxS184L.** *bak^−/−^bax^−/−^* MEFs stably expressing the indicated Bak or Bax variants, were also transfected with pMIH (IRES-Hygro) retroviral vector expressing FLAG-Bcl-x_L_ or FLAG-Mcl-1. After selection by serial passage in hygromycin, polyclonal populations were lysed with 1% Triton X-100 prior to immunoprecipitation with anti-FLAG affinity resin (Sigma). Immunoprecipitated samples and cell lysates were then immunoblotted for FLAG, Bak or Bax. Results are representative of two independent experiments.
(TIF)

## References

[1] Lindsten T, Ross AJ, King A, Zong W, Rathmell JC (2000). The combined functions of proapoptotic Bcl-2 family members Bak and Bax are essential for normal development of multiple tissues.. Mol Cell.

[2] Wei MC, Zong WX, Cheng EH, Lindsten T, Panoutsakopoulou V (2001). Proapoptotic BAX and BAK: a requisite gateway to mitochondrial dysfunction and death.[see comment].. Science.

[3] Antonsson B, Montessuit S, Sanchez B, Martinou JC (2001). Bax is present as a high molecular weight oligomer/complex in the mitochondrial membrane of apoptotic cells.. Journal of Biological Chemistry.

[4] Eskes R, Desagher S, Antonsson B, Martinou JC (2000). Bid induces the oligomerization and insertion of Bax into the outer mitochondrial membrane.. Molecular and Cellular Biology.

[5] Griffiths GJ, Corfe BM, Savory P, Leech S, Esposti MD (2001). Cellular damage signals promote sequential changes at the N-terminus and BH-1 domain of the pro-apoptotic protein Bak.. Oncogene.

[6] Wei MC, Lindsten T, Mootha VK, Weiler S, Gross A (2000). tBID, a membrane-targeted death ligand, oligomerizes BAK to release cytochrome c.. Genes Dev.

[7] Westphal D, Dewson G, Czabotar PE, Kluck RM (2011). Molecular biology of Bax and Bak activation and action.. Biochim Biophys Acta 1813.

[8] Dewson G, Kratina T, Czabotar P, Day CL, Adams JM (2009). Bak Activation for Apoptosis Involves Oligomerization of Dimers via Their alpha6 Helices.. Mol Cell.

[9] Dewson G, Kratina T, Sim HW, Puthalakath H, Adams JM (2008). To trigger apoptosis Bak exposes its BH3 domain and homo-dimerizes via BH3:grooove interactions.. Mol Cell.

[10] Oh KJ, Singh P, Lee K, Foss K, Lee S (2010). Conformational changes in BAK, a pore-forming proapoptotic Bcl-2 family member, upon membrane insertion and direct evidence for the existence of BH3-BH3 contact interface in BAK homo-oligomers.. J Biol Chem.

[11] Bleicken S, Classen M, Padmavathi PV, Ishikawa T, Zeth K (2010). Molecular details of Bax activation, oligomerization, and membrane insertion.. J Biol Chem.

[12] Dewson G, Ma S, Frederick P, Hockings C, Tan I (2011). Bax dimerizes via a symmetric BH3:groove interface during apoptosis.. Cell Death Differ.

[13] George NM, Evans JJ, Luo X (2007). A three-helix homo-oligomerization domain containing BH3 and BH1 is responsible for the apoptotic activity of Bax.. Genes Dev.

[14] Zhang Z, Zhu W, Lapolla SM, Miao Y, Shao Y (2010). Bax forms an oligomer via separate, yet interdependent, surfaces.. J Biol Chem.

[15] Dewson G, Kluck RM (2009). Mechanisms by which Bak and Bax permeabilise mitochondria during apoptosis.. J Cell Sci.

[16] Gavathiotis E, Suzuki M, Davis ML, Pitter K, Bird GH (2008). BAX activation is initiated at a novel interaction site.. Nature.

[17] Kim H, Tu HC, Ren D, Takeuchi O, Jeffers JR (2009). Stepwise activation of BAX and BAK by tBID, BIM, and PUMA initiates mitochondrial apoptosis.. Mol Cell.

[18] Kuwana T, Bouchier-Hayes L, Chipuk JE, Bonzon C, Sullivan BA (2005). BH3 Domains of BH3-Only Proteins Differentially Regulate Bax-Mediated Mitochondrial Membrane Permeabilization Both Directly and Indirectly.. Mol Cell.

[19] Letai A, Bassik MC, Walensky LD, Sorcinelli MD, Weiler S (2002). Distinct BH3 domains either sensitize or activate mitochondrial apoptosis, serving as prototype cancer therapeutics.. Cancer Cell.

[20] Willis SN, Chen L, Dewson G, Wei A, Naik E (2005). Proapoptotic Bak is sequestered by Mcl-1 and Bcl-xL, but not Bcl-2, until displaced by BH3-only proteins.. Genes & Development.

[21] Llambi F, Moldoveanu T, Tait SW, Bouchier-Hayes L, Temirov J (2011). A unified model of mammalian BCL-2 protein family interactions at the mitochondria.. Mol Cell.

[22] Smits C, Czabotar PE, Hinds MG, Day CL (2008). Structural plasticity underpins promiscuous binding of the prosurvival protein A1.. Structure.

[23] Willis SN, Fletcher JI, Kaufmann T, van Delft MF, Chen L (2007). Apoptosis initiated when BH3 ligands engage multiple Bcl-2 homologs, not Bax or Bak.[see comment].. Science.

[24] Gillissen B, Essmann F, Graupner V, Starck L, Radetzki S (2003). Induction of cell death by the BH3-only Bcl-2 homolog Nbk/Bik is mediated by an entirely Bax-dependent mitochondrial pathway.. EMBO Journal.

[25] Wang GQ, Wieckowski E, Goldstein LA, Gastman BR, Rabinovitz A (2001). Resistance to granzyme B-mediated cytochrome *c* release in Bak-deficient cells.. Journal of Experimental Medicine.

[26] Shimazu T, Degenhardt K, Nur EKA, Zhang J, Yoshida T (2007). NBK/BIK antagonizes MCL-1 and BCL-XL and activates BAK-mediated apoptosis in response to protein synthesis inhibition.. Genes & Development.

[27] Schinzel A, Kaufmann T, Schuler M, Martinalbo J, Grubb D (2004). Conformational control of Bax localization and apoptotic activity by Pro168.. Journal of Cell Biology.

[28] Setoguchi K, Otera H, Mihara K (2006). Cytosolic factor- and TOM-independent import of C-tail-anchored mitochondrial outer membrane proteins.. EMBO Journal.

[29] Dewson G, Kratina T, Sim HW, Puthalakath H, Adams JM (2008). To trigger apoptosis Bak exposes its BH3 domain and homo-dimerizes via BH3:grooove interactions.. Molecular Cell.

[30] Nechushtan A, Smith CL, Hsu YT, Youle RJ (1999). Conformation of the Bax C-terminus regulates subcellular location and cell death.. EMBO Journal.

[31] Valentijn AJ, Upton JP, Gilmore AP (2008). Analysis of endogenous Bax complexes during apoptosis using blue native PAGE: implications for Bax activation and oligomerization.. Biochem J.

[32] Borgese N, Colombo S, Pedrazzini E (2003). The tale of tail-anchored proteins: coming from the cytosol and looking for a membrane.. Journal of Cell Biology.

[33] Horie C, Suzuki H, Sakaguchi M, Mihara K (2002). Characterization of signal that directs C-tail-anchored proteins to mammalian mitochondrial outer membrane.. Molecular Biology of the Cell.

[34] Ausili A, Torrecillas A, Martinez-Senac MM, Corbalan-Garcia S, Gomez-Fernandez JC (2008). The interaction of the Bax C-terminal domain with negatively charged lipids modifies the secondary structure and changes its way of insertion into membranes.. J Struct Biol.

[35] Martinez-Senac Mdel M, Corbalan-Garcia S, Gomez-Fernandez JC (2002). The structure of the C-terminal domain of the pro-apoptotic protein Bak and its interaction with model membranes.. Biophysical Journal.

[36] Wolter KG, Hsu YT, Smith CL, Nechushtan A, Xi XG (1997). Movement of Bax from the cytosol to mitochondria during apoptosis.. Journal of Cell Biology.

[37] Edlich F, Banerjee S, Suzuki M, Cleland MM, Arnoult D (2011). Bcl-x(L) Retrotranslocates Bax from the Mitochondria into the Cytosol.. Cell.

[38] Suzuki M, Youle RJ, Tjandra N (2000). Structure of Bax: coregulation of dimer formation and intracellular localization.. Cell.

[39] Kaufmann T, Schlipf S, Sanz J, Neubert K, Stein R (2003). Characterization of the signal that directs Bcl-x(L), but not Bcl-2, to the mitochondrial outer membrane.. Journal of Cell Biology.

[40] Oliver L, Priault M, Tremblais K, LeCabellec M, Meflah K (2000). The substitution of the C-terminus of bax by that of bcl-xL does not affect its subcellular localization but abrogates its pro-apoptotic properties.. FEBS Letters.

[41] Uren RT, Dewson G, Chen L, Coyne SC, Huang DCS (2007). Mitochondrial permeabilization relies on BH3 ligands engaging multiple pro-survival Bcl-2 relatives, not Bak.. J Cell Biol.

[42] Nechushtan A, Smith CL, Hsu YT, Youle RJ (1999). Conformation of the Bax C-terminus regulates subcellular location and cell death.. EMBO J.

[43] Suzuki M, Youle RJ, Tjandra N (2000). Structure of Bax: coregulation of dimer formation and intracellular localization.. Cell.

[44] Fletcher JI, Meusburger S, Hawkins CJ, Riglar DT, Lee EF (2008). Apoptosis is triggered when prosurvival Bcl-2 proteins cannot restrain Bax.. Proc Natl Acad Sci U S A.

[45] Annis MG, Soucie EL, Dlugosz PJ, Cruz-Aguado JA, Penn LZ (2005). Bax forms multispanning monomers that oligomerize to permeabilize membranes during apoptosis.. EMBO Journal.

[46] Chen L, Willis SN, Wei A, Smith BJ, Fletcher JI (2005). Differential targeting of prosurvival Bcl-2 proteins by their BH3-only ligands allows complementary apoptotic function.. Molecular Cell.

[47] Dai H, Smith A, Meng XW, Schneider PA, Pang YP (2011). Transient binding of an activator BH3 domain to the Bak BH3-binding groove initiates Bak oligomerization.. J Cell Biol.

[48] Mason KD, Carpinelli MR, Fletcher JI, Collinge JE, Hilton AA (2007). Programmed anuclear cell death delimits platelet life span.. Cell.

[49] Moldoveanu T, Liu Q, Tocilj A, Watson MH, Shore G (2006). The x-ray structure of a BAK homodimer reveals an inhibitory zinc binding site.. Molecular Cell.

[50] Day CL, Smits C, Fan FC, Lee EF, Fairlie WD (2008). Structure of the BH3 domains from the p53-inducible BH3-only proteins Noxa and Puma in complex with Mcl-1.. J Mol Biol.

[51] Liu X, Dai S, Zhu Y, Marrack P, Kappler JW (2003). The structure of a Bcl-xL/Bim fragment complex: implications for Bim function.. Immunity.

