# Supplementary figures and images for: Translocation of a Bak C-Terminus Mutant from Cytosol to Mitochondria to Mediate Cytochrome c Release: Implications for Bak and Bax Apoptotic Function
Source: PLoS One. 2012 Mar 19;7(3):e31510. doi: 10.1371/journal.pone.0031510 (PMC3307716; doi:10.1371/journal.pone.0031510)

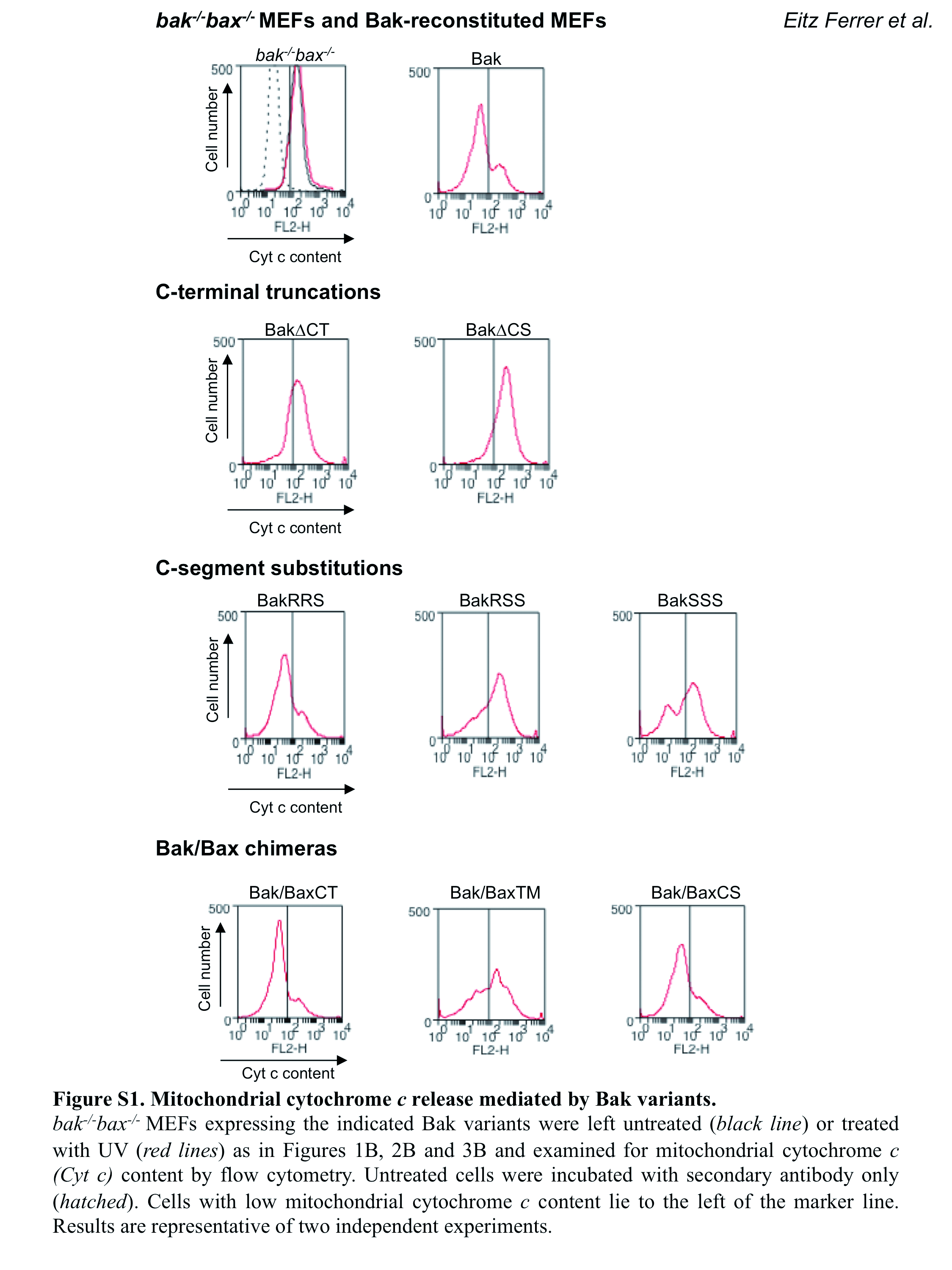

Supplement: Figure S1 — Mitochondrial cytochrome c release mediated by Bak variants. bak−/−bax−/− MEFs expressing the indicated Bak variants were left untreated (black line) or treated with UV (red lines) as in Figures 1B, 2B and 3B and examined for mitochondrial cytochrome c (Cyt c) content by flow cytometry (Waterhouse and Trapani, Cell Death Differ 2003 Jul; 10 (7): 853–855) using anti-cytochrome c antibody (6H2.B4; Pharmingen) and R-phycoerythrin-conjugated secondary antibody (1031-09; Southern Biotech, AL, USA). Untreated cells were incubated with secondary antibody only (hatched). Cells with low mitochondrial cytochrome c content lie to the left of the marker line. Results are representative of two independent experiments. (TIF) [file pone.0031510.s001.tif]

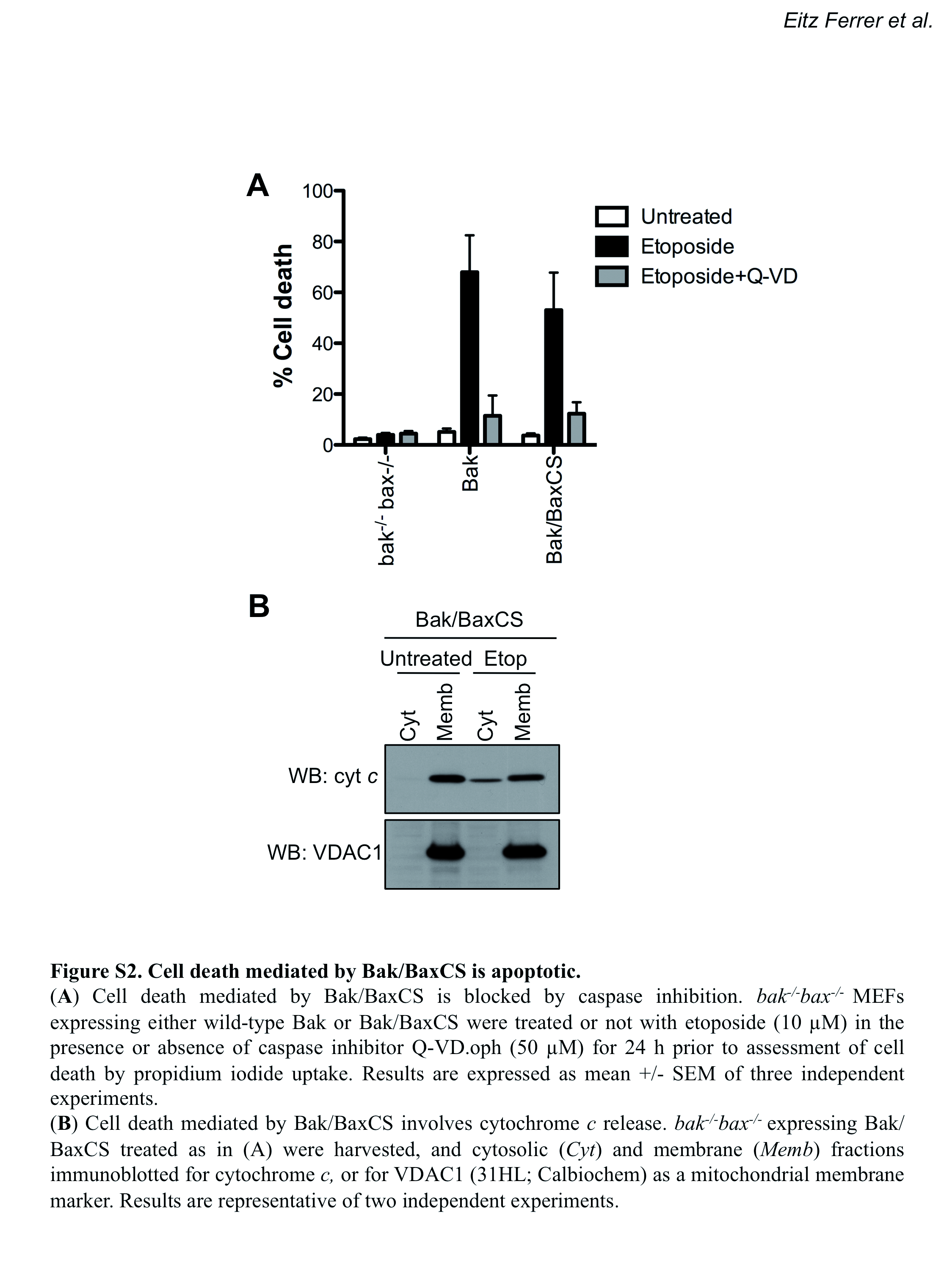

Supplement: Figure S2 — Cell death mediated by Bak/BaxCS is apoptotic. (A) Cell death mediated by Bak/BaxCS is blocked by caspase inhibition. bak−/−bax−/− MEFs expressing either wild-type Bak or Bak/BaxCS were treated or not with etoposide (10 µM) in the presence or absence of caspase inhibitor Q-VD.oph (50 µM) for 24 h prior to assessment of cell death by propidium iodide uptake. Results are expressed as mean +/− SEM of three independent experiments. (B) Cell death mediated by Bak/BaxCS involves cytochrome c release. bak−/−bax−/− expressing Bak/BaxCS treated as in (A) were harvested, and cytosolic (Cyt) and membrane (Memb) fractions immunoblotted for cytochrome c, or for VDAC1 (31HL; Calbiochem) as a mitochondrial membrane marker. Results are representative of two independent experiments. (TIF) [file pone.0031510.s002.tif]

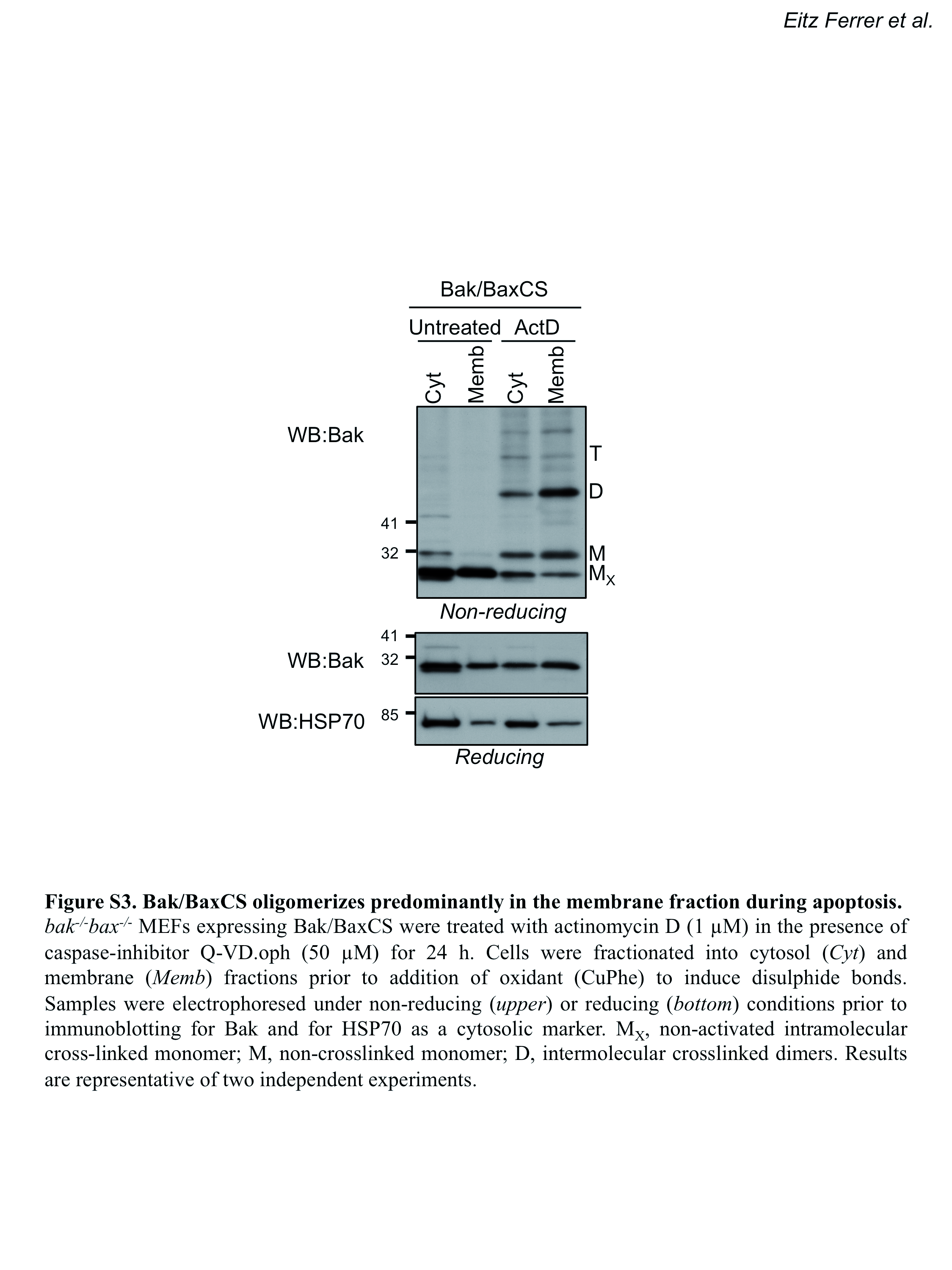

Supplement: Figure S3 — Bak/BaxCS oligomerizes predominantly in the membrane fraction during apoptosis. bak−/−bax−/− MEFs expressing Bak/BaxCS were treated with actinomycin D (1 µM) in the presence of caspase-inhibitor Q-VD.oph (50 µM) for 24 h. Cells were fractionated into cytosol (Cyt) and membrane (Memb) fractions prior to addition of oxidant (CuPhe) to induce disulphide bonds. Samples were electrophoresed under non-reducing (upper) or reducing (bottom) conditions prior to immunoblotting for Bak and for HSP70 as a cytosolic marker. MX, non-activated intramolecular cross-linked monomer; M, non-crosslinked monomer; D, intermolecular crosslinked dimers. Results are representative of two independent experiments. (TIF) [file pone.0031510.s003.tif]

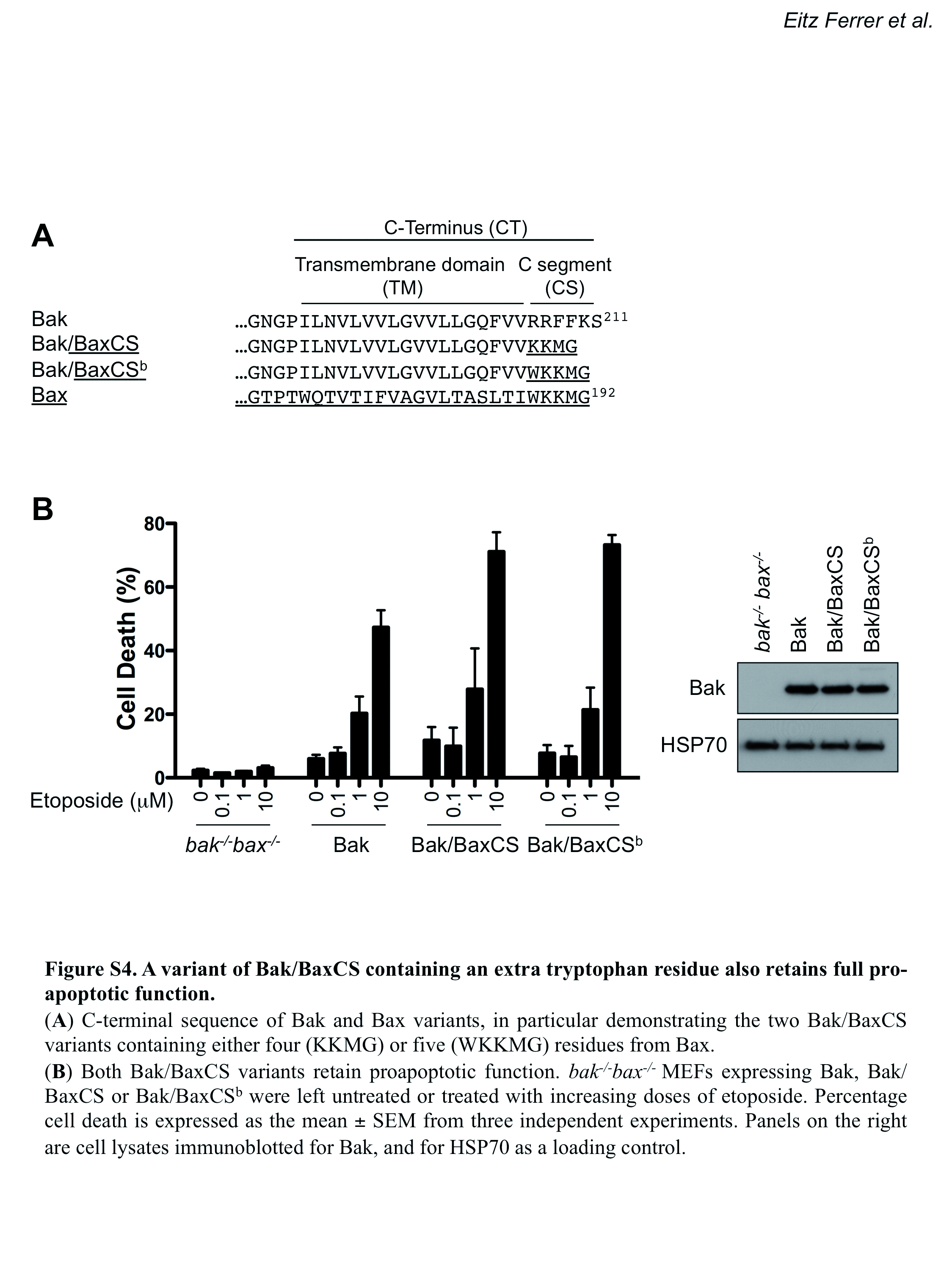

Supplement: Figure S4 — A variant of Bak/BaxCS containing an extra tryptophan residue also retains full pro-apoptotic function. (A) C-terminal sequence of Bak and Bax variants, in particular demonstrating the two Bak/BaxCS variants containing either four (KKMG) or five (WKKMG) residues from Bax. (B) Both Bak/BaxCS variants retain proapoptotic function. bak−/−bax−/− MEFs expressing Bak, Bak/BaxCS or Bak/BaxCSb were left untreated or treated with increasing doses of etoposide. Percentage cell death is expressed as the mean ± SEM from three independent experiments. Statistical significance for the 10 mM dose when compared to Bak is shown; *p<0.05. Panels on the right are cell lysates immunoblotted for Bak, and for HSP70 as a loading control. (TIF) [file pone.0031510.s004.tif]

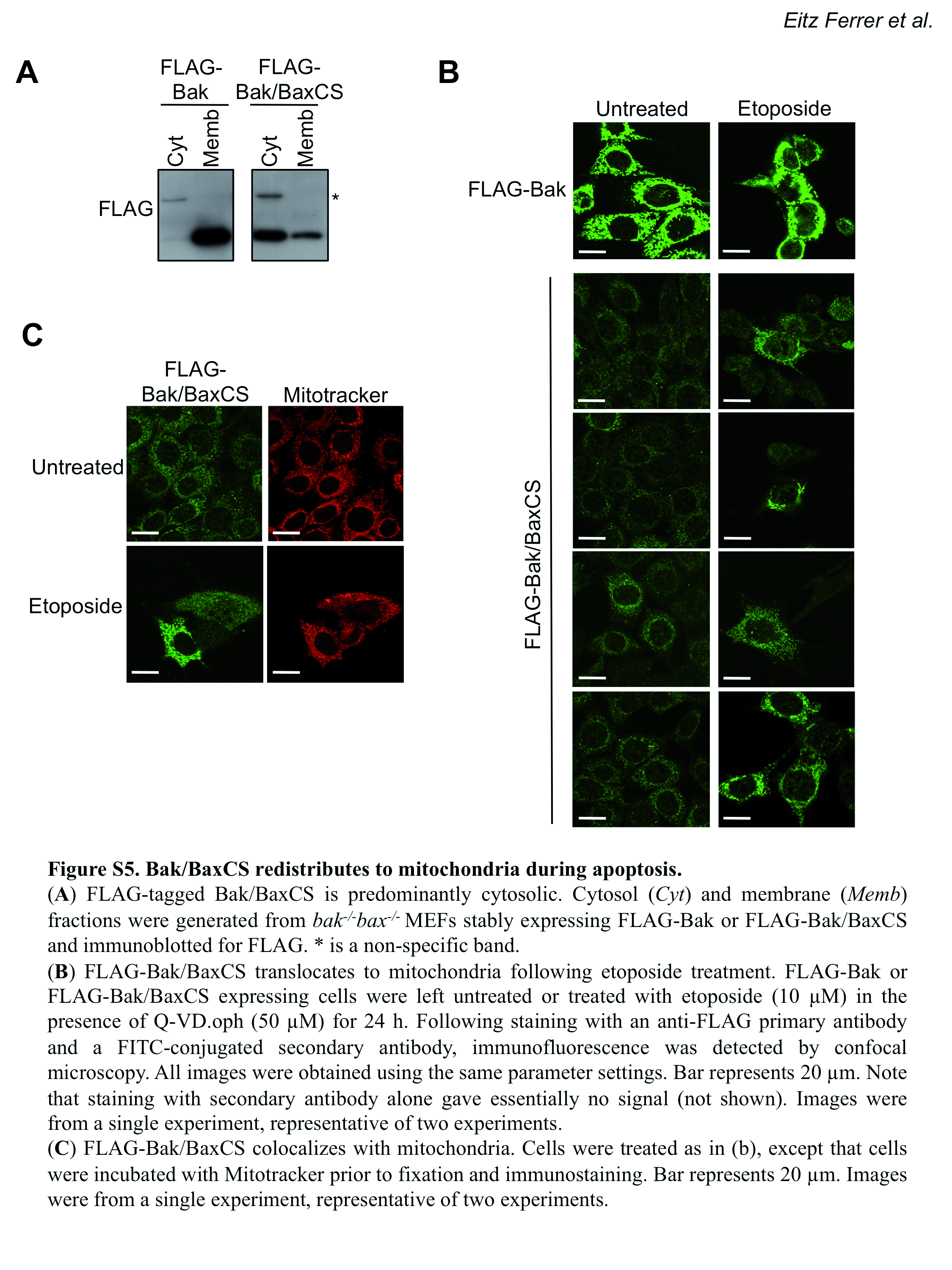

Supplement: Figure S5 — Bak/BaxCS redistributes to mitochondria during apoptosis. (A) FLAG-tagged Bak/BaxCS is predominantly cytosolic. Cytosol (Cyt) and membrane (Memb) fractions were generated from bak−/−bax−/− MEFs stably expressing FLAG-Bak or FLAG-Bak/BaxCS and immunoblotted for FLAG. * is a non-specific band. (B) FLAG-Bak/BaxCS translocates to mitochondria following etoposide treatment. FLAG-Bak or FLAG-Bak/BaxCS expressing cells were left untreated or treated with etoposide (10 µM) in the presence of Q-VD.oph (50 µM) for 24 h, and fixed in 4% paraformaldehyde for 20 mins at room temperature. Following staining with an anti-FLAG primary antibody (M2; Sigma) and FITC-conjugated secondary antibody (Southern Biotech, AL, USA), immunofluorescence was detected by confocal microscopy (Leica SP2). All images were obtained using the same parameter settings. Bar represents 20 µm. Note that staining with secondary antibody alone gave essentially no signal (not shown). Images were from a single experiment, representative of two experiments. (C) FLAG-Bak/BaxCS colocalizes with mitochondria. Cells were treated as in (B), except that cells were incubated with with MitoTracker Deep Red FM (0.5 µM; Invitrogen, CA) for 30 mins at 37°C prior to fixation and immunostaining. Bar represents 20 µm. Images were from a single experiment, representative of two experiments. (TIF) [file pone.0031510.s005.tif]

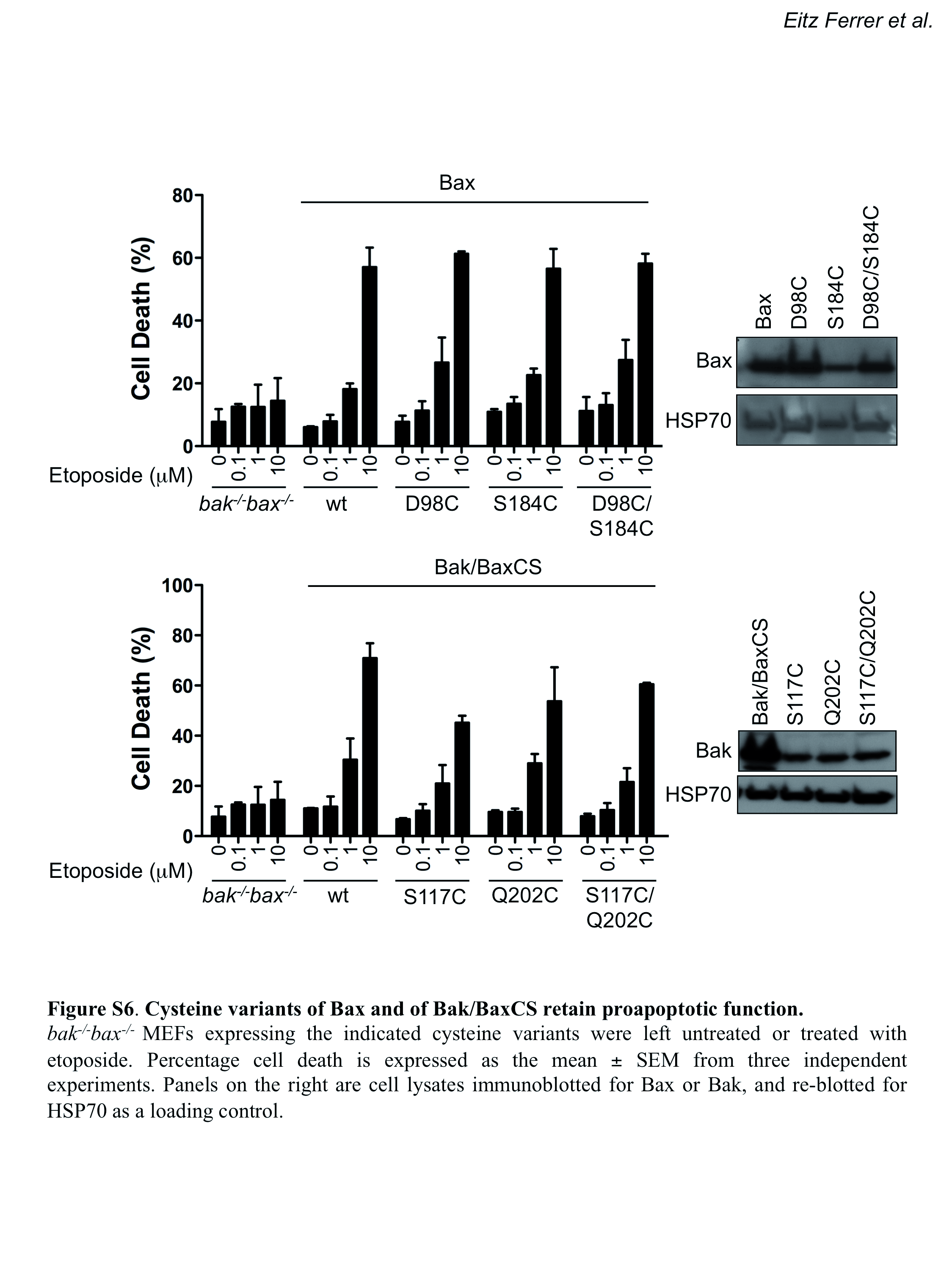

Supplement: Figure S6 — Cysteine variants of Bax and of Bak/BaxCS retain proapoptotic function. bak−/−bax−/− MEFs expressing the indicated cysteine variants were left untreated or treated with etoposide. Percentage cell death is expressed as the mean ± SEM from three independent experiments. Statistical significance for the 10 mM dose when compared to wild-type (wt) protein is shown; *p<0.05. Panels on the right are cell lysates immunoblotted for Bax or Bak, and re-blotted for HSP70 as a loading control. (TIF) [file pone.0031510.s006.tif]

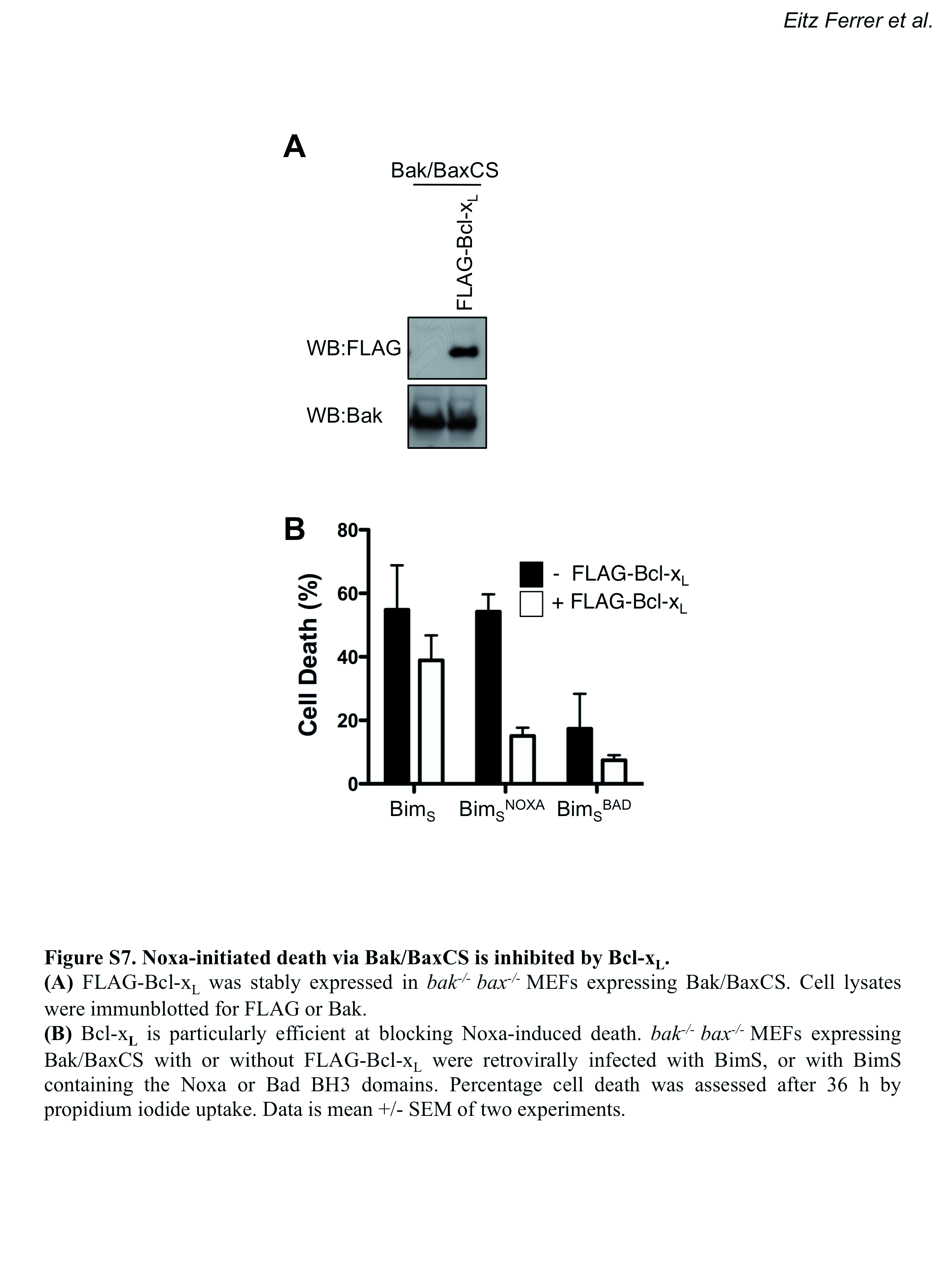

Supplement: Figure S7 — Noxa-initiated death via Bak/BaxCS is inhibited by Bcl-xL. (A) FLAG-Bcl-xL was stably expressed in bak−/−bax−/− MEFs expressing Bak/BaxCS. Cell lysates were immunblotted for FLAG or Bak. (B) Bcl-xL is particularly efficient at blocking Noxa-induced death. bak−/−bax−/− MEFs expressing Bak/BaxCS with or without FLAG-Bcl-xL were retrovirally infected with BimS, or with BimS containing the Noxa or Bad BH3 domains. Percentage cell death was assessed after 36 h by propidium iodide uptake. Data is mean +/− SEM of two experiments. Statistical significance for the effect of Bcl-xL is shown; *p<0.05. (TIF) [file pone.0031510.s007.tif]

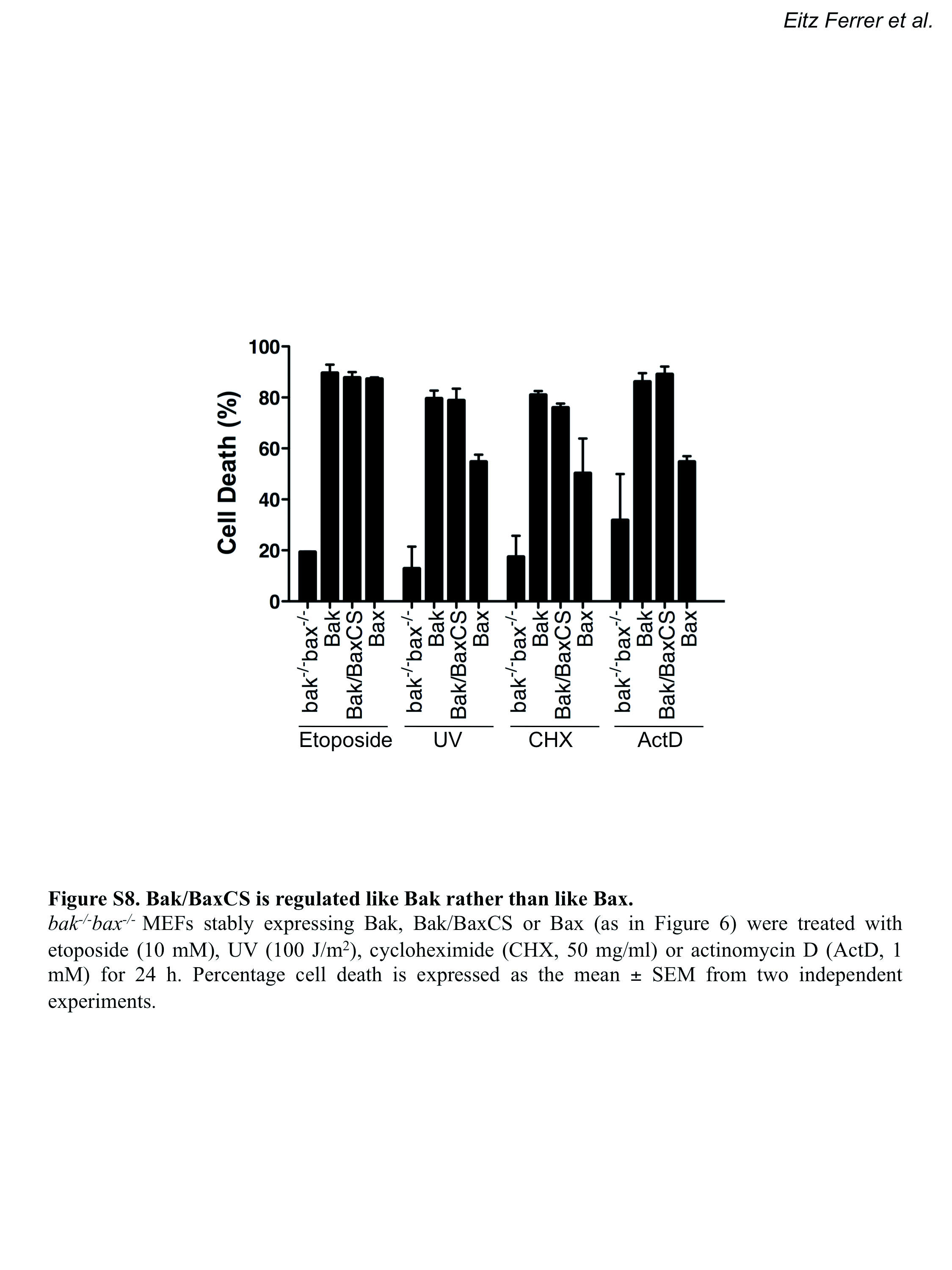

Supplement: Figure S8 — Bak/BaxCS is regulated like Bak rather than like Bax. bak−/−bax−/− MEFs stably expressing Bak, Bak/BaxCS or Bax (as in Figure 6) were treated with etoposide (10 mM), UV (100 J/m2), cycloheximide (CHX, 50 mg/ml) or actinomycin D (ActD, 1 mM) for 24 h. Percentage cell death is expressed as the mean ± SEM from two independent experiments. Statistical significance for each variant compared to Bak is shown; *p<0.05. (TIF) [file pone.0031510.s008.tif]

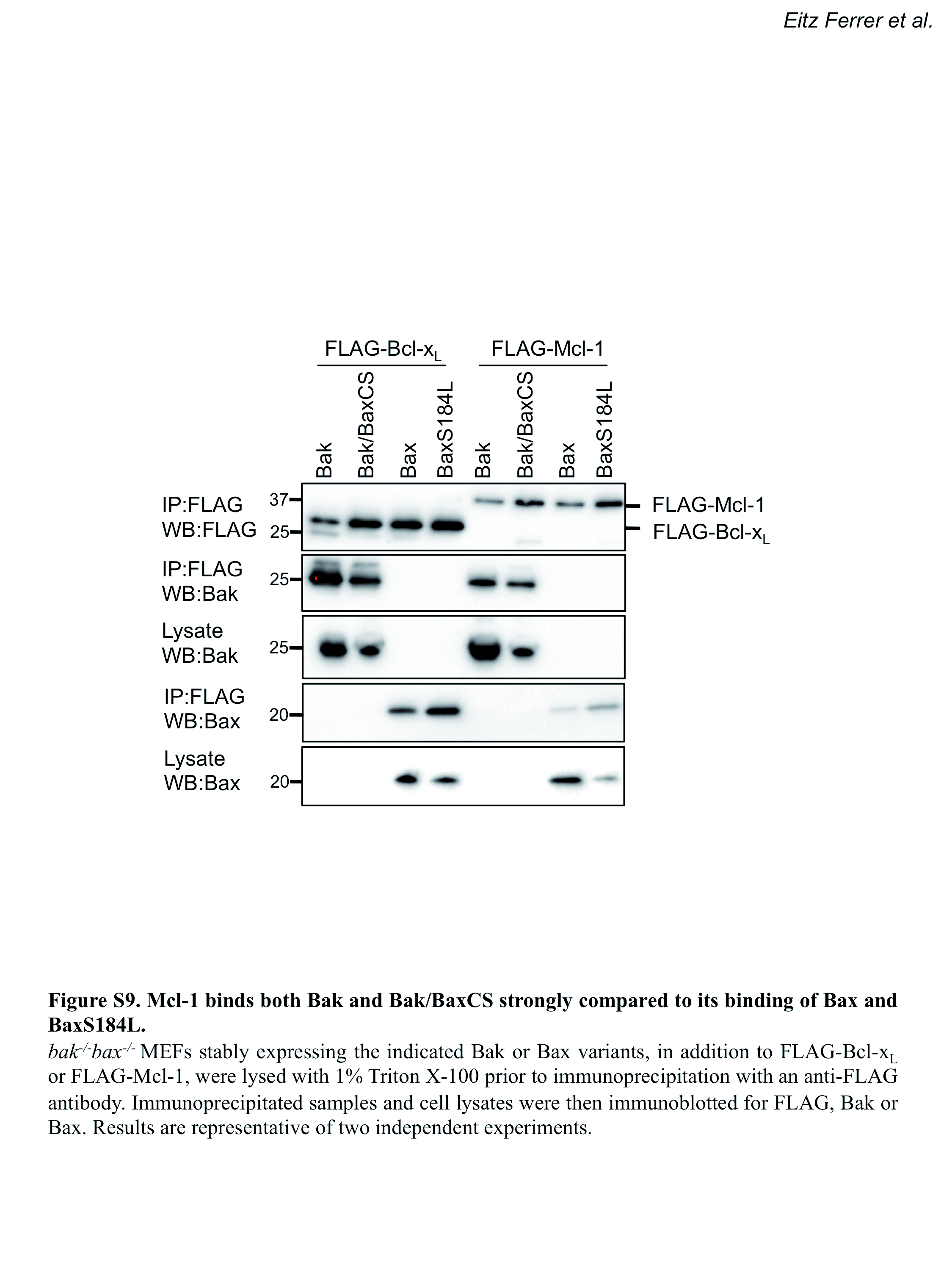

Supplement: Figure S9 — Mcl-1 binds both Bak and Bak/BaxCS strongly compared to its binding of Bax and BaxS184L. bak−/−bax−/− MEFs stably expressing the indicated Bak or Bax variants, were also transfected with pMIH (IRES-Hygro) retroviral vector expressing FLAG-Bcl-xL or FLAG-Mcl-1. After selection by serial passage in hygromycin, polyclonal populations were lysed with 1% Triton X-100 prior to immunoprecipitation with anti-FLAG affinity resin (Sigma). Immunoprecipitated samples and cell lysates were then immunoblotted for FLAG, Bak or Bax. Results are representative of two independent experiments. (TIF) [file pone.0031510.s009.tif]
